# *In vivo* Hypoxia and a Fungal Alcohol Dehydrogenase Influence the Pathogenesis of Invasive Pulmonary Aspergillosis

**DOI:** 10.1371/journal.ppat.1002145

**Published:** 2011-07-21

**Authors:** Nora Grahl, Srisombat Puttikamonkul, Jeffrey M. Macdonald, Michael P. Gamcsik, Lisa Y. Ngo, Tobias M. Hohl, Robert A. Cramer

**Affiliations:** 1 Department of Immunology and Infectious Diseases, Montana State University, Bozeman, Montana, United States of America; 2 Joint Department of Biomedical Engineering, University of North Carolina, Chapel Hill, and North Carolina State University, Raleigh, North Carolina, United States of America; 3 Infectious Disease Sciences, Vaccine and Infectious Disease Division, Clinical Research Division, Fred Hutchinson Cancer Research Center, Seattle, Washington, United States of America; David Geffen School of Medicine at University of California Los Angeles, United States of America

## Abstract

Currently, our knowledge of how pathogenic fungi grow in mammalian host environments is limited. Using a chemotherapeutic murine model of invasive pulmonary aspergillosis (IPA) and ^1^H-NMR metabolomics, we detected ethanol in the lungs of mice infected with *Aspergillus fumigatus*. This result suggests that *A. fumigatus* is exposed to oxygen depleted microenvironments during infection. To test this hypothesis, we utilized a chemical hypoxia detection agent, pimonidazole hydrochloride, in three immunologically distinct murine models of IPA (chemotherapeutic, X-CGD, and corticosteroid). In all three IPA murine models, hypoxia was observed during the course of infection. We next tested the hypothesis that production of ethanol *in vivo* by the fungus is involved in hypoxia adaptation and fungal pathogenesis. Ethanol deficient *A. fumigatus* strains showed no growth defects in hypoxia and were able to cause wild type levels of mortality in all 3 murine models. However, lung immunohistopathology and flow cytometry analyses revealed an increase in the inflammatory response in mice infected with an alcohol dehydrogenase null mutant strain that corresponded with a reduction in fungal burden. Consequently, in this study we present the first *in vivo* observations that hypoxic microenvironments occur during a pulmonary invasive fungal infection and observe that a fungal alcohol dehydrogenase influences fungal pathogenesis in the lung. Thus, environmental conditions encountered by invading pathogenic fungi may result in substantial fungal metabolism changes that influence subsequent host immune responses.

## Author Summary

Metabolic flexibility is important for human pathogens like *Aspergillus fumigatus* as it allows adaptation to dynamic infection induced microenvironments. Consequently, identification of fungal metabolic pathways critical for *in vivo* growth may uncover novel virulence mechanisms and new therapeutic opportunities. To date, the mechanisms used by *A. fumigatus* to adapt to microenvironments in immunosuppressed mammalian hosts are poorly understood. In this study we discover that *A. fumigatus* is exposed to oxygen limiting microenvironments during invasive pulmonary aspergillosis (IPA). Thus, this result builds on growing evidence that suggests hypoxia is a significant *in vivo* stress encountered by human fungal pathogens. We tested the hypothesis that genes encoding enzymes involved in ethanol fermentation are important for *in vivo* fungal responses to hypoxia. We consequently observed a significant increase in the inflammatory response that correlated with reduced fungal growth in the lungs of mice inoculated with an alcohol dehydrogenase null mutant. Altogether, our study suggests that fungal responses to *in vivo* hypoxic microenvironments can directly affect host immune responses to the invading fungal pathogen. A better understanding of these mechanisms will increase our understanding of IPA and other human diseases caused by fungi and could potentially lead to improved therapeutic options.

## Introduction

The incidence of life-threatening human fungal infections has increased during the last three decades as medical therapies, organ transplantations, an increasing geriatric population, and HIV infections have generated a significant rise in the number of susceptible patients [1], [2], [3]. *Aspergillus fumigatus*, a commonly encountered mold found in soil and organic debris [4], [5], is responsible for a number of clinically relevant diseases in immunocompromised and immunocompetent individuals. Among these, invasive pulmonary aspergillosis (IPA) is the most lethal with mortality rates ranging from 30–90% depending on the patient population [6], [7], [8], [9], [10], [11].

To cause lethal disease, *A. fumigatus* must face and overcome a number of *in vivo* microenvironment challenges once it is inhaled into the lower respiratory tract. However, our understanding of the dynamic microenvironments encountered by the fungus in the mammalian lung, and the mechanisms by which it grows in these microenvironments, are poorly understood. Arguably, understanding the mechanisms by which *A. fumigatus* is able to grow in the mammalian host environment will lead to either improvement of existing therapeutic options or development of novel treatments through the identification of novel drug targets. Some of the previously studied environmental factors encountered by *A. fumigatus* during *in vivo* growth include: high temperature, changes in pH, oxidative stress, and a restricted nutrient supply. In all probability, these stresses are similar to those that the mold has to overcome to be a highly competitive member of the compost microflora, and subsequently it has evolved multi-faceted and robust mechanisms to overcome these challenges [12], [13], [14], [15], [16].

An important characteristic of *A. fumigatus*'*s* saprophytic lifestyle that has largely been overlooked is its ability to adapt to a wide range of oxygen levels. *Aspergillus* species are generally considered obligate aerobes, but *A. fumigatus* has been observed to tolerate oxygen levels as low as 0.1% [17], [18]. In compost piles, oxygen concentrations range from atmospheric (21%) to hypoxic (1.5% and lower) and rapidly change with the metabolic activity of the compost microflora [19]. Thus, organisms such as *A. fumigatus* that thrive in such environments likely have evolved mechanisms to tolerate hypoxia. With regard to fungal-human interactions, oxygen availability in mammalian tissues is also substantially below atmospheric levels [20], [21], [22]. Even in the alveoli of healthy lungs, the most aerated organ and primary site of *Aspergillus* deposition, the oxygen level is around 14%. By the time oxygen reaches the capillaries and diffuses into surrounding tissues its availability is much lower with levels of 2–4% reported [23], [24]. Thus, microorganisms that colonize, inhabit and infect mammalian hosts are subject to dynamic ranges of oxygen availability depending on their location in the mammalian body. Moreover, the collateral effects of microbial infections, inflammation, thrombosis, and necrosis, are often thought to decrease available oxygen concentrations even further [25], [26], [27], [28]. However, the occurrence and effects of hypoxia on the outcome of human fungal infections, especially those that primarily occur in the lung, are poorly understood [29], [30].

Recent evidence supporting the hypothesis that hypoxia is a significant component of fungal pathogenesis comes from studies on the sterol-regulatory element binding protein (SREBP) transcription factor in *Cryptococcus neoformans* and *A. fumigatus*. Null mutants of the respective SREBP in each fungal pathogen were incapable of growth in hypoxic conditions and subsequently were also avirulent in murine models of cryptococcosis and IPA [31], [32], [33]. Though the virulence defect in these SREBP mutants may be caused by other pleiotropic factors, their ability to grow in normoxic but not hypoxic conditions strongly suggests that adaptation and growth in hypoxia are contributing factors to the avirulence of these strains. Yet, as mentioned, whether hypoxia occurs during an invasive fungal infection in commonly used murine models of fungal disease is unknown.

In this study, we observe for the first time that hypoxic microenvironments do occur in three immunologically distinct murine models of IPA. We also observe that a key gene, which encodes an enzyme required for the last step of ethanol fermentation in response to hypoxia, influences IPA pathogenesis through modulation of the inflammatory response. Thus, we conclude that *in vivo* hypoxic microenvironments do occur during IPA and that fungal responses to these conditions can influence fungal pathogenesis. These results lay the foundation for further studies to identify how human pathogenic fungi adapt to hypoxia and how these adaptation mechanisms ultimately influence the outcome of fungal pathogenesis in mammals.

## Results

### *In vivo* ethanol production by *A. fumigatus* in a murine model of invasive pulmonary aspergillosis

In order to gain an understanding of the important metabolic pathways utilized by *Aspergillus fumigatus* during growth in the mammalian lung, we examined qualitative production of metabolites in a chemotherapeutic murine model of invasive pulmonary aspergillosis (IPA) utilizing broncheoalveolar lavages (BAL) and ^1^H-NMR. Our chemotherapeutic model of IPA is characterized by the use of cyclophosphamide and the corticosteroid triamcinolone to induce immunosuppression. Visual inspection of the BAL sample spectra taken from uninfected control mice and mice inoculated with *A. fumigatus* on day +3 post-inoculation revealed a relatively small number of identifiable metabolites and few differences. Identifiable metabolites in all samples included taurine, choline, creatine, acetate, and lactate (Figure S1). Given the low complexity of BAL samples that are predominately 0.7% saline, this result is not surprising. Surprisingly, however, ethanol was detected in 4 of the 10 mice infected with *A. fumigatus,* but in none of the uninfected controls (Figure S1). Attempts to detect ethanol at later time points during infection using BALs or lung homogenates were not successful. Thus, the extent of ethanol production *in vivo* during IPA remains to be determined.

**Figure 1:**
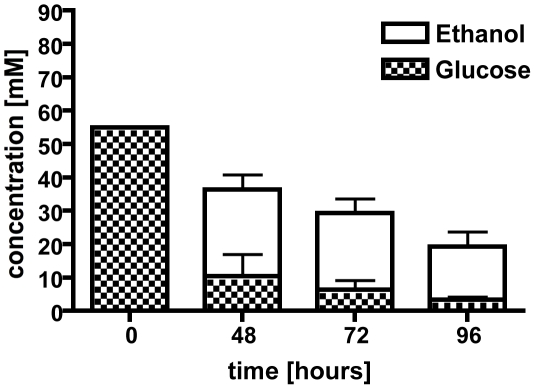
Culture supernatants were used in a high performance liquid chromatography (HPLC) analysis to determine ethanol production and glucose consumption. After 48 hours under hypoxic conditions, ethanol was detected along with a decrease in glucose concentration. Ethanol concentrations decrease over time with a peak at 48 hours. Each value represents mean ± standard error (n = 2 independent cultures). *,**,#p<0.05.

However, to support the hypothesis that the ethanol production was fungal in origin, we next tested whether *A. fumigatus* was capable of fermenting glucose to ethanol *in vitro* in glucose minimal media (1% glucose) under normoxic or hypoxic conditions. While no ethanol was detectable after 24 h in either condition, we could detect ethanol in the culture supernatants after 48, 72, and 96 h of growth in hypoxia (1% oxygen, Figure 1). The glucose levels expectedly dropped during the time course of the shake flask cultures, from 55 mM at time zero to less than 4 mM at 96 h, and this corresponded with a decrease in detectable ethanol (Figure 1). Fermentation is associated with hypoxic or anoxic environments in many organisms including plant pathogenic fungi, Crabtree negative yeast, pathogenic bacteria, and even plants [34], [35], [36], [37], [38], [39], [40]. We thus hypothesized that *A. fumigatus* encounters hypoxic or anoxic microenvironments during IPA.

### Hypoxia occurs in murine models of invasive pulmonary aspergillosis

To test the hypothesis that *A. fumigatus* encounters hypoxia during IPA, we used a hypoxia marker, pimonidazole hydrochloride, a nitroheterocyclic drug whose hypoxia-dependent activation by cellular mammalian nitroreductases (severe hypoxia: 10 mmHg partial oxygen pressure, ≤1% oxygen) leads to the formation of covalent intracellular adducts with thiol groups on proteins, peptides, amino acids, and the drug itself [41], [42], [43], [44]. The resulting protein adducts are effective immunogens and can be used to “visualize” hypoxia *in vivo* with immunofluorescence.

**Figure 2:**
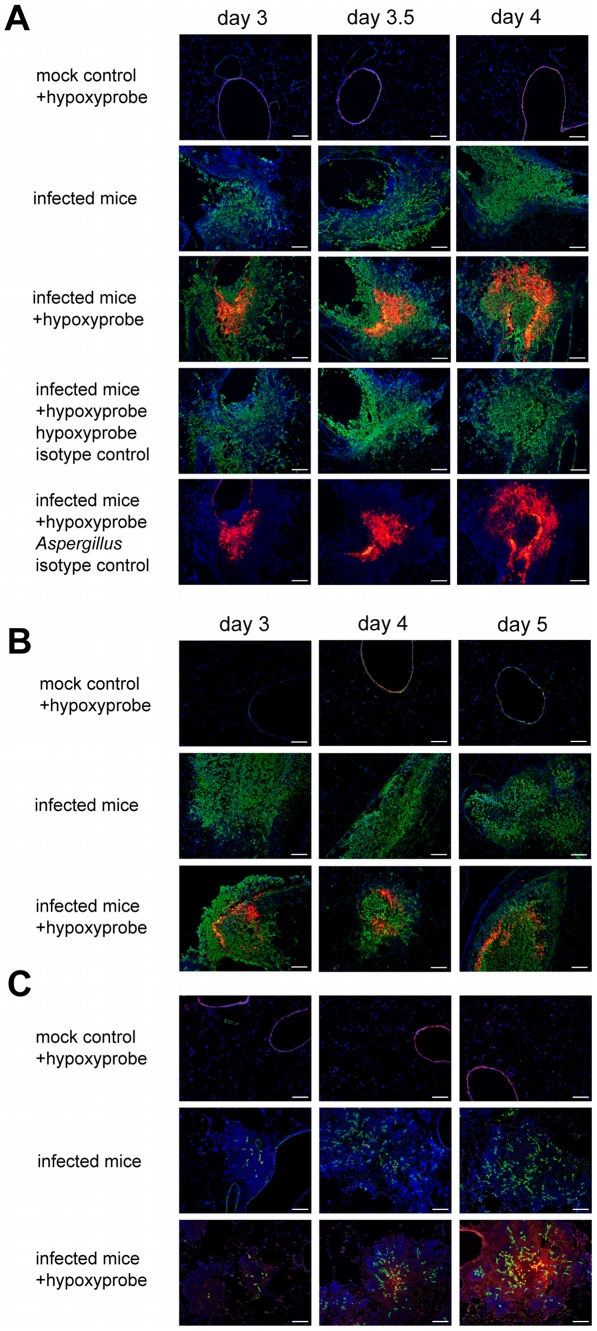
Identification of hypoxic microenvironments at the site of *A. fumigatus* infections in murine lungs. The following three distinct mouse IPA models were utilized: (**A**) triamcinolone (corticosteroid) model, (**B**) chemotherapy model, and (**C**) X-CGD mouse model. Mice were euthanized on indicated days after inoculation (mock control = 0.01% Tween inoculated, infected mice = inoculated with *A. fumigatus* strain CEA10). Prior to sacrifice, Hypoxyprobe-1 (60 mg/kg) was intravenously injected into indicated mice (+hypoxyprobe) and allowed to circulate for 60 to 90 min. After tissue preparation specific antibodies were used to detect Hypoxyprobe-1 bound to proteins (red) and *A. fumigatus* hyphae (green). Isotype controls were only stained with either one of the secondary antibodies to verify specific staining. Host cell nuclei were counterstained with DAPI (blue). Merged pictures show co-localization of the fungal hyphae (green), surrounding immune cells (blue), and Hypoxyprobe-1 (red) in all three IPA mouse models. Isotype controls and infected mice without Hypoxyprobe-1 injection show that no unspecific staining occurred. Bar = 100 µm.

We tested for the development of hypoxia *in vivo* in three immunologically distinct murine models of IPA (chemotherapeutic, corticosteroid, and X-CGD) (Figure 2). Each model represents a different clinically relevant mechanism of immunosuppression. As mentioned, the chemotherapeutic model attempts to reproduce the immunological state of severely immunosuppressed patients who have often undergone a bone marrow transplant. This model is characterized by a severe depletion of neutrophils and other important immune effector cells needed to prevent and control invasive fungal infections. Another patient population highly susceptible to invasive fungal infections is those patients on high doses of corticosteroids for treatment of graft versus host disease or other autoimmune type diseases. In our model of this patient population, we utilized a single high dose of the corticosteroid triamcinolone. Unlike the chemotherapeutic model, this model is not characterized by depletion of effector cells, but rather by a suppression of their antifungal activity that leads to altered inflammatory responses. Finally, we utilized transgenic mice that are deficient in the gp91 Phox subunit of the NADPH oxidase. These mice are a close model for the genetic disorder chronic granulomatous disease (CGD), and are highly susceptible to *Aspergillus* infection.

In the Triamcinolone model, histopathology of *A. fumigatus* inoculated mice show lesions with a strong influx of immune cells (blue) and strong growth of fungal hyphae (green) invading into the lung parenchyma from the airways (Figure 2A, Figure S2). By day 3 p.i., hypoxia could readily be detected in the center of larger lesions (red), and while the hypoxic areas of the lesions are comparable on day 3.5 p.i., they are significantly expanded on day 4 p.i.. The isotype control staining of the same lesions in subsequent tissue sections, as well as complete staining of tissue sections from inoculated mice without hypoxyprobe injections, demonstrate the specificity of the hypoxyprobe and antibodies utilized (Figure 2A).

In contrast to the Triamcinolone model, lesions in the chemotherapeutic model are dominated by massive fungal growth causing significant tissue necrosis with minimal inflammation (Figure 2B, Figure S2). Given the severe neutropenia associated with this model, this result is expected. Despite the strong reduction in the inflammatory response and extensive fungal growth in this model, we were able to detect hypoxia in these lesions at similar time points to the Triamcinolone model (Figure 2A and B). However, it is clear that the amount and extent of hypoxia is significantly reduced in this murine model.

In mice that lack the gp91^phox^ component of NADPH oxidase (a model of X-CGD) the lesion size gradually increased during the time course of infection from day 3 to day 5, which was due to a strong increase in the inflammatory response of the host [45]. Fungal growth in this model was strongly reduced in comparison to the other two tested murine models (Figure 2C, Figure S2). On day 3 p.i. minimal amounts of hypoxia were detected, but by day 4 p.i. significant levels of hypoxia were observed in the center of the lesions. On day 5 p.i. hypoxia was abundantly present in the center of the lesions, and throughout the surrounding tissue indicating that significant parts of the lung experience hypoxia in this murine model of IPA (Figure 2C). Indeed, in some animals in this model almost the entire lung seemed hypoxic at later time points just prior to mortality (data not shown). Taken together, these data confirm that *A. fumigatus* encounters hypoxic microenvironments (oxygen concentrations ≤1%) and a dynamic range of oxygen availability during murine models of IPA. The extent of hypoxia, fungal growth, and host immune responses in the different models suggests that the host inflammatory response plays an important, but not exclusive, role in the generation of the hypoxic microenvironment.

### Generation and characterization of ethanol fermentation deficient mutants

Given the evidence that *A. fumigatus* encounters hypoxia during IPA and that production of ethanol occurs *in vivo* during infection and is normally used by microbes to survive in low oxygen environments [34], [35], [36], [37], [38], [39], we next tested the hypothesis that ethanol fermentation was a key mechanism for hypoxia adaptation and fungal virulence.

To determine the effects of ethanol fermentation on IPA pathogenesis, we searched the *A. fumigatus* genome sequence for genes encoding enzymes known to be involved in ethanol fermentation [46], [47]. Using the *A. nidulans pdcA* (pyruvate decarboxylase) (*An_pdcA –* AN4888) gene sequence for a BLASTX search of the *A. fumigatus* genome (CADRE genome database) we identified three potential candidates that may encode for pyruvate decarboxylases and named them *pdcA* (AFUB_038070: 85% identity to *An_pdcA*), *pdcB* (AFUB_096720: 39% identity to *An_pdcA*), and *pdcC* (AFUB_062480: 33% identity to *An_pdcA*) (Table 1). Protein sequence analysis (InterProScan Sequence Search, http://www.ebi.ac.uk/Tools/InterProScan/) suggested all three *A. fumigatus* proteins were pyruvate decarboxylases (Table 1). In a similar manner, the known alcohol dehydrogenase (Adh) gene sequences of *A. nidulans* (AdhI: *An*_*alcA –* AN8979, AdhII: *An_alcB –* AN3741, and AdhIII: *An*_*alcC –* AN2286) were used to identify the most likely genes encoding alcohol dehydrogenases in *A. fumigatus*. However, as the *Aspergillus fumigatus* genome contains several predicted alcohol dehydrogenases we restricted our search to proteins with high identity and similarity to the *A. nidulans* Adh proteins (*alcA* – AFUB_087590: 87% identity and 94% similarity to *An_alcA*, *alcB* – AFUB_089920: 79% identity and 89% similarity to *An_alcB*, and *alcC* – AFUB_053780: 80% identity and 91% similarity to *An_alcC*) (Table 1).

**Table 1:** Genes involved in *A. fumigatus* ethanol fermentation pathway.

Gene ID	Gene Name	Query Gene	Similarity	Identity	Domains	Enzyme
**AFUB_038070**	**pdcA**	**AN_pdcA**	**93%**	**85%**	C-terminal TPP-binding, central domain, N-terminal TPP-binding domain	Pyruvate decarboxylase
**AFUB_096720**	**pdcB**	**AN_pdcA**	**57%**	**39%**	C-terminal TPP-binding, central domain, N-terminal TPP-binding domain	Pyruvate decarboxylase
**AFUB_062480**	**pdcC**	**AN_pdcA**	**47%**	**33%**	TPP-binding enzyme conserved site, C-terminal TPP-binding, central domain, N-terminal TPP-binding domain	Pyruvate decarboxylase
**AFUB_087590**	**alcA**	**AN_alcA**	**94%**	**87%**	zinc-containing conserved site, GroES-like domain, ADH C-terminal domain, ADH GroES-like domain, NAD(P)-binding domain	Alcohol Dehydrogenase
**AFUB_089920**	**alcB**	**AN_alcB**	**89%**	**79%**	zinc-containing conserved site, GroES-like domain, ADH C-terminal domain, ADH GroES-like domain, NAD(P)-binding domain	Alcohol Dehydrogenase
**AFUB_053780**	**alcC**	**AN_alcC**	**91%**	**80%**	zinc-containing conserved site, GroES-like domain, ADH C-terminal domain, ADH GroES-like domain, NAD(P)-binding domain	Alcohol Dehydrogenase

AN = Aspergillus nidulans.

TPP = Thiamine pyrophosphate.

ADH = alcohol dehydrogenase.

**Figure 3:**
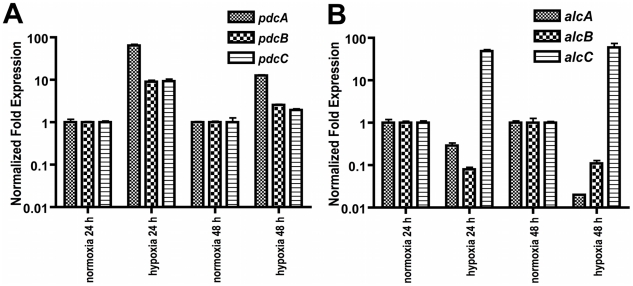
Normalized fold expression of *pdc* genes and *alc* genes in *A. fumigatus* under normoxic and hypoxic conditions. (**A**) mRNA levels of all three *pdc* genes increase in hypoxia with *pdcA* being the most responsive. mRNA levels of the *pdc* genes show a reduction in hypoxia over time. (**B**) Of the three tested *alc* genes only mRNA from *alcC* is increased in response to hypoxia. Quantification of mRNA was achieved by quantitative real-time PCR. Values are normalized to β–tubulin and shown relative to normoxia. Results are the mean and standard deviation of three replicates.

Given the findings that *A. fumigatus* utilizes ethanol fermentation in *in vitro* and possibly *in vivo* hypoxic environments and the apparent gene redundancy in the predicted ethanol fermentation pathway, we next sought to determine which of the genes transcriptionally responds to hypoxia. Quantitative real-time PCR comparing the mRNA abundance of the ethanol fermentation genes under hypoxic and normoxic conditions revealed an immediate increase in mRNA abundance of all three *pdc* genes as well as the *alcC* gene to hypoxic growth conditions (Figure 3). While mRNA abundance levels of *pdcB* and *pdcC* show a ∼9-fold higher normalized fold expression after 24 h in hypoxia, *pdcA* mRNA showed a 64-fold increase compared to normoxic culture conditions. This data suggest that PdcA is the primary pyruvate decarboxylase that responds to hypoxia in *A. fumigatus*. With regard to the alcohol dehydrogenase encoding genes, the mRNA abundance of *alcC* significantly increased in response to hypoxia while mRNA abundance of the other two alcohol dehydrogenase encoding genes did not (Figure 3). These data suggest that *alcC* is the primary gene encoding an alcohol dehydrogenase that responds to hypoxia in *A. fumigatus*.

**Figure 4:**
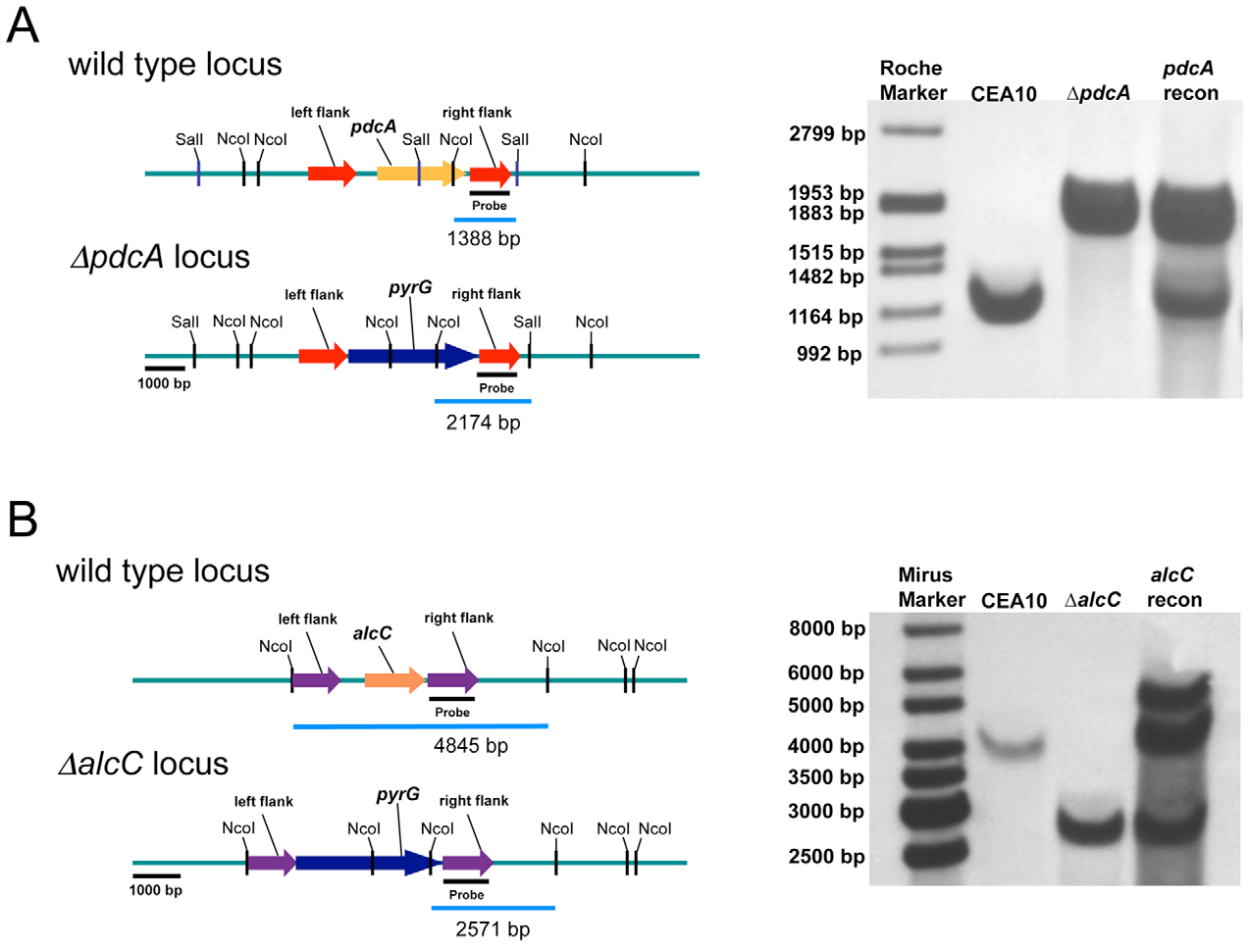
Generation and confirmation of PdcA and AlcC null mutants in *A. fumigatus*. Schematic of wild type (CEA10), PdcA (**A**), and AlcC (**B**) null mutant genomic loci. (**A**) Southern blot analysis of wild type, *ΔpdcA*, and *pdcA* recon strains as well as (**B**) wild type, *ΔalcC*, and *alcC* recon strains. Genomic DNA from the respective strains was isolated and digested overnight with SalI and NcoI in the case of PdcA and with only NcoI for AlcC. An approximate 1 kb genomic region was utilized as a probe. The expected hybridization patterns and sizes were observed for all strains tested. In addition, confirmation of ectopic reconstitution was confirmed by the presence of the wild type locus hybridization signal and persistence of the null mutant locus. The *alcC* reconstituted strain showed a double re-insertion of the *alcC* locus.

**Figure 5:**
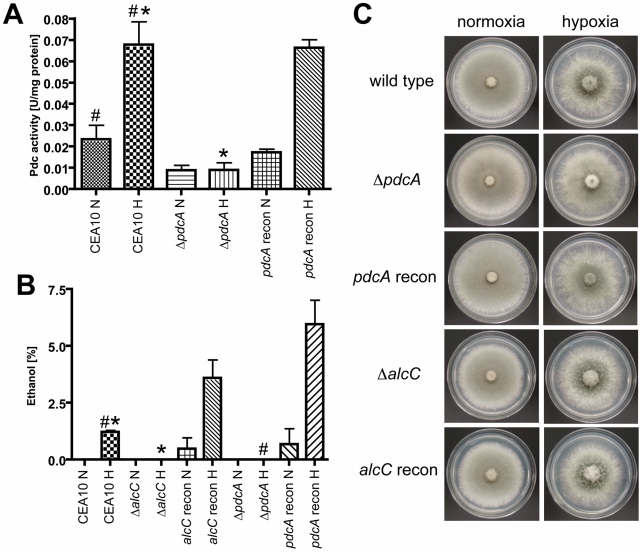
PdcA and AlcC are required for *in vitro* ethanol fermentation but not hypoxic growth of *A. fumigatus*. (**A**) After 48 hours in hypoxia, mycelia were harvested and pyruvate decarboxylase activity (Pdc) of cell free extracts was determined. Compared to CEA10, the *ΔpdcA* strain showed a ∼80% reduction of Pdc activity under hypoxic conditions, which could be restored in the reconstituted strain (*,# p<0.01) (**B**) Ethanol concentration in culture supernatants was determined with GCMS, showing that PdcA and AlcC are required for ethanol production of *A. fumigatus*. Each value represents mean ± standard error (n = 3 for each condition for each strain). (*,# p<0.01). **(C)** 1x10^6^ conidia of CEA10, *ΔpdcA*, *pdcA* recon, *ΔalcC*, and *alcC* recon were plated on GMM (glucose) plates and incubated at 37°C for 48 h under normoxic or hypoxic conditions. All strains showed comparable growth and morphology in all tested conditions.

To determine whether these genes are involved in ethanol fermentation, we generated null mutants of the genes encoding PdcA, PdcB, PdcC, and AlcC by replacement of the coding sequence in *A. fumigatus* strain CEA17 with the auxotrophic marker pyrG from *A. parasiticus* (Figure 4 and data not shown). A *pdcA*/*pdcB* double mutant was also generated. Ectopic re-introduction of the wild type *pdcA* and *alcC* allele into *ΔpdcA* and *ΔalcC* (resulting in strains *pdcA* recon and *alcC* recon) allowed us to attribute all resulting phenotypes specifically to the absence of *pdcA* or *alcC*. The genotype of all strains was confirmed with PCR analyses (data not shown) and Southern blots (Figure 4 and data not shown). Southern blot analysis of the *alcC* recon strain revealed a double insertion of the *alcC* encoding sequence in the genome and the *alcC* recon strain displayed a 10-fold higher mRNA abundance in response to hypoxia as the *alcC* allele in the wild type strain (data not shown). However, the double insertion had no detectable phenotypic effect on the reconstituted strain, since the *alcC* recon strain showed the same phenotype as the wild type in all further experiments with only a slight but statistically insignificant increase in ethanol production (Figure 5B).

Next, we examined the ability of the generated null mutant strains to produce ethanol in response to *in vitro* hypoxic growth conditions. The loss of *pdcA* decreases Pdc enzyme activity in hypoxia by approximately 80% (Figure 5A) and the activity can be restored to wild type levels in the *pdcA* recon strain. The *ΔpdcB* and *ΔpdcC*, as well as the *ΔalcC* strain showed no significant decrease in Pdc activity (data not shown), confirming the hypothesis that *pdcA* is the most important *pdc* gene in *A. fumigatus* for production of ethanol, at least *in vitro*. However there is still residual activity detectable in the Δ*pdcA* and the *ΔpdcA*/*ΔpdcB* strains (0.0047 ± 0.0041 in normoxia and 0.0039 ± 0.0064 in hypoxia; data not shown). A triple mutant of all three putative PDC encoding genes would need to be generated to definitively answer whether the observed residual activity from the cell free extracts is indeed real Pdc activity.

Using the culture supernatants from the above experiments we examined the amount of ethanol produced by the respective fungal strains. The *pdcB* and *pdcC* null mutant strains show a slight but statistically insignificant decrease in ethanol production that is essentially similar to wild type levels (CEA10: 0.071±0.035%; Δ*pdcB*: 0.047±0.002; Δ*pdcC*: 0.040±0.006; P>0.4). Importantly, no ethanol could be detected in *ΔpdcA* and *ΔalcC* culture supernatants, while the wild type and respective reconstituted strains produced ethanol (Figure 5B). These results support the gene expression and Pdc enzyme activity assays that suggest PdcA is the primary Pdc and that AlcC is the primary alcohol dehydrogenase required for *in vitro* ethanol production in *A. fumigatus*. The function of the remaining Pdc and Alc genes in *A. fumigatus* thus is not currently clear.

To determine whether ethanol fermentation is important for growth under hypoxic conditions, we examined radial growth on solid media under normoxic and hypoxic conditions. As previously described, *A. fumigatus* grows well under hypoxic conditions on the fermentable carbon source glucose [18], [31] (Figure 5C). Surprisingly, the ethanol fermentation deficient mutants show no growth defect on glucose containing media under hypoxic (1% or 0.2% O_2_ (data not shown)) conditions compared to the wild type and the reconstituted strains (Figure 5C). In addition, the wild type and mutant strains are all also able to grow on the non-fermentable carbon sources ethanol, lactate and glycerol, although the growth rate is decreased compared to growth on glucose (data not shown). Germination rates were the same for all strains utilized and no defects in conidia viability were observed (data not shown). Liquid biomass quantification with the respective mutant strains also revealed no growth differences between wild type and ethanol deficient strains in hypoxia (data not shown). Taken together, these results suggest that PdcA and AlcC are the primary enzymes involved in ethanol fermentation in *A. fumigatus,* but that other unidentified mechanisms are utilized to grow under hypoxic conditions on fermentable carbon sources when ethanol fermentation is not possible.

### Deletion of alcohol dehydrogenase III in *A. fumigatus* alters the pathogenesis of invasive pulmonary aspergillosis

**Figure 6:**
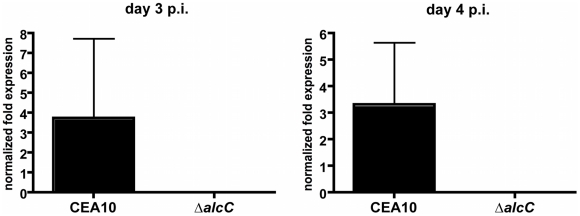
Normalized fold expression of the *A. fumigatus alcC* gene *in vivo* during fungal pathogenesis. Outbred CD1 mice were immunosuppressed by single injection of Triamcinolone (40 mg/kg) 1 day prior to *A. fumigatus* intranasal inoculation. Lungs were harvested on indicated days and whole RNA was prepared. Quantification of mRNA was achieved by quantitative real-time PCR using fungal specific primers. Values are normalized to *tefA* and shown relative to mock control animals. Results are the mean and standard deviation of three replicates.

**Figure 7:**
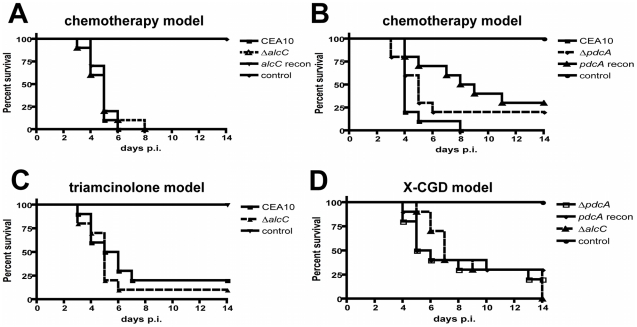
Loss of ethanol fermentation does not affect survival of *A. fumigatus* infected mice. (**A**) (**B**) Outbred CD-1 mice were immunosuppressed by intraperitoneal (i.p.) injection of 175 mg/kg cyclophosphamide 2 days prior to infection and 150 mg/kg 3 days post-inoculation, and subcutaneous (s.c.) injection of Triamcinolone (40 mg/kg) 1 day prior to infection and 6 days post-infection. Mice were inoculated with ∼10^4^ conidia of the indicated strains by inhalation of an aerosol in a Hinner's chamber. (**C**) For the triamcinolone model outbred CD-1 mice were immunosuppressed on day -1 by s.c. injection of Kenalog (40 mg/kg), followed by inhalation-inoculation with 10^4^ conidia on day 0. (**D**) gp91^phox−/−^ mice were challenged intranasally with 10^6^ conidia in a volume of 25 µl of the indicated strains. The ethanol deficient mutant strains, *ΔalcC* and *ΔpdcA*, showed no difference in mortality compared to the wild type or reconstituted strains in any of the tested IPA models (p>0.2, Log-Rank Test). 10 animals were inoculated per experiment per inoculation group and the experiments were repeated in duplicate.

Despite the general lack of a pathogenesis associated phenotype of the *in vitro* ethanol production deficient strains, ethanol itself has been observed to have significant immunomodulatory properties [48], [49], [50], [51], [52]. In addition, utilizing quantitative real-time PCR we found that *alcC* is expressed *in vivo* during fungal pathogenesis on day 3 and 4 post inoculation in the triamcinolone model (Figure 6) suggesting that this gene and the enzyme it encodes may be important for *in vivo* growth. Therefore, we sought to determine the effects of loss of PdcA and AlcC on the pathogenesis of IPA. We first examined the virulence of the Δ*pdcA* and Δ*alcC* strains in the chemotherapeutic and X-linked chronic granulomatous disease (X-CGD, gp91^phox−/−^ mice) murine models of IPA [45], [53]. Irrespective of the fungal strain, *A. fumigatus* infected mice, in both models, displayed well described symptoms of *A. fumigatus* infection including hunched posture, ruffled fur, weight loss, and increased respiration as early as day +2 of inoculation. Subsequently, no difference in mortality was observed between the null mutant *(ΔalcC, ΔpdcA,* and Δ*pdcA*Δ*pdcB*) and wild type strains (Figure 7 and data not shown).

**Figure 8:**
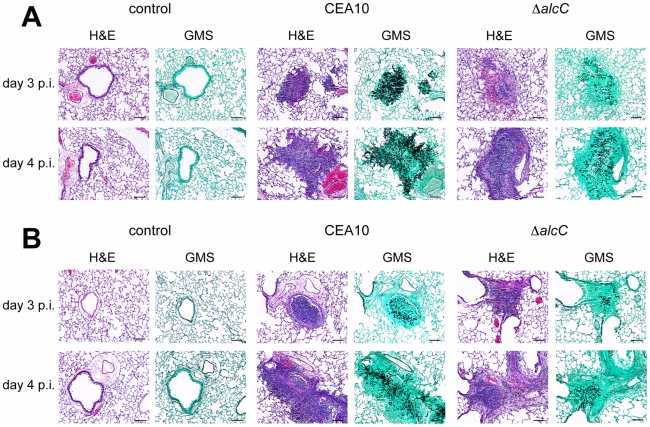
Representative histopathology of the chemotherapy and triamcinolone mouse models 3 and 4 days after inoculation. Mice were inoculated with 1x10^4^ conidia by inhalation (Control  = 0.01% Tween inoculated), euthanized on indicated days, lungs removed, fixed, and stained with hematoxylin and eosin (H&E) or Gommori's methenamine silver (GMS) stain. (**A**) In the chemotherapy IPA model wild type inoculated mice show substantial fungal growth and a strongly reduced influx of immune cells, while lesions of Δ*alcC* inoculated mice show reduced fungal growth and an increased inflammatory response on day 3 and day 4 p.i.. (**B**) Lung histopathology of triamcinolone model mice showed reduced fungal growth of the wild type with robust inflammation compared to (A) and an even further reduction in growth of Δ*alcC* with an apparent increase in inflammation and cellular influx. Bar = 100 µm.

To further examine the impact of the mutant strains on the pathogenesis of IPA in these murine models, lung histopathology was performed. Lungs from X-CGD mice displayed the expected histopathology for this model including a large inflammatory response with reduced fungal growth (Figure S3). In this model, histopathology of wild type and Δ*alcC* inoculated mice looked identical at all time points examined (Figure S3). In the chemotherapy model, pulmonary lesions of wild type infected animals show substantial fungal growth and invasion of the lung parenchyma with a minimal influx of immune cells but extensive tissue necrosis, hemorrhaging, edema, and tissue damage (Figure 8A). Importantly, mice inoculated with Δ*alcC* show lesions with reduced fungal growth and more inflamed tissue compared to wild type inoculated mice (despite the overall lesion sizes being comparable between the two inoculation groups) (Figure 8A). Taken together, this result suggests that AlcC plays a potential role in the pathogenesis of IPA.

To explore this hypothesis further, we utilized the Triamcinolone (corticosteroid) model of IPA. IPA in mice treated with corticosteroids have previously been observed to induce hyper-inflammatory responses that are speculated to be the primary cause of mortality in this model [54]. Thus, we rationalized that any changes in the inflammatory response to *A. fumigatus* in the absence of AlcC would be potentiated in this model. As in the chemotherapeutic and X-CGD murine models, Δ*alcC* infected animals displayed wild type levels of mortality in the Triamcinolone model (Figure 7C). A similar change in gross histopathology of the Triamcinolone compared to the chemotherapeutic model infected with the *ΔalcC* strain is also observed (Figure 8B). Consistent with the observations in the chemotherapy model, Δ*alcC* inoculated animals show less fungal growth but increased levels of inflammation (Figure 8B).

**Figure 9:**
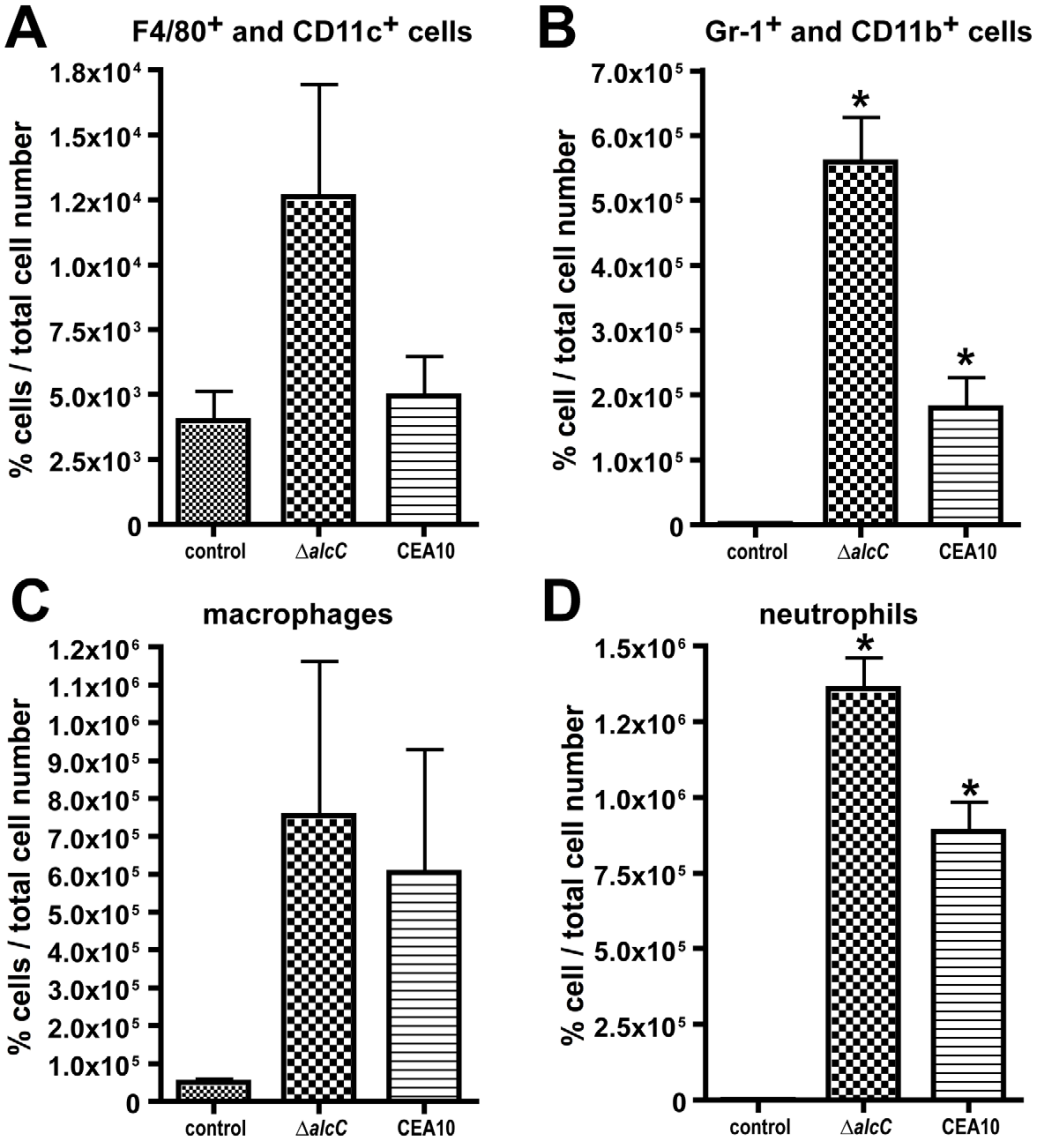
Characterization of cellular infiltrates in broncheoalveolar (BAL) fluids of triamcinolone immunosuppressed mice. Flow cytometry was used to identify (**A**) F4/80^+^/CD11c^+^ and (**B**) GR-1^+^/CD11b^+^ cells. Figures S3 and S4 show the corresponding Dot plots. Both cell types are elevated in Δ*alcC* inoculated mice. In agreement, differential cell counts demonstrate that macrophage and monocyte numbers were slightly increased in mice inoculated with Δ*alcC* (**C**) while neutrophil numbers were significantly elevated (**D**). *p<0.05. Results are presented as mean and standard error of N = 5 mice. The experiment was repeated in duplicate with similar results.

Altogether these observations suggest that loss of AlcC results in an increased inflammatory response to *A. fumigatus*. To further characterize and quantify these histopathology observations, we analyzed the cellular infiltrates in BAL fluids of Triamcinolone treated mice from 2 different inoculation experiments using flow cytometry and differential cell counts. On day 3 p.i., Δ*alcC* inoculated mice show an increased quantity of F4/80^+^/CD11c^+^ cells and a significant increase in GR-1^+^/CD11b^+^ cells in the BAL fluids compared to wild type infected animals (Figure 9A and B, and Figure S4 and S5). F4/80^+^/CD11c^+^ cells most likely represent macrophages while GR-1^+^/CD11b^+^ cells are most likely neutrophils. As expected, control mice BALs contained F4/80^+^/CD11c^+^ cells but nearly no GR-1^+^/CD11b^+^ cells. Furthermore, differential cell counts of BAL fluids revealed that macrophages, monocytes and particularly neutrophils were the dominant cell types found in the BAL samples of *A. fumigatus* inoculated mice. Consistent with the observed histopathology as well as the flow cytometry data, differential cell count numbers of neutrophils were significantly increased in Δ*alcC* inoculated mice (p<0.05; Figure 9C and D). Taken together, these data support the histopathology observations that indicate an increased inflammatory response in mice infected with Δ*alcC*.

**Figure 10:**
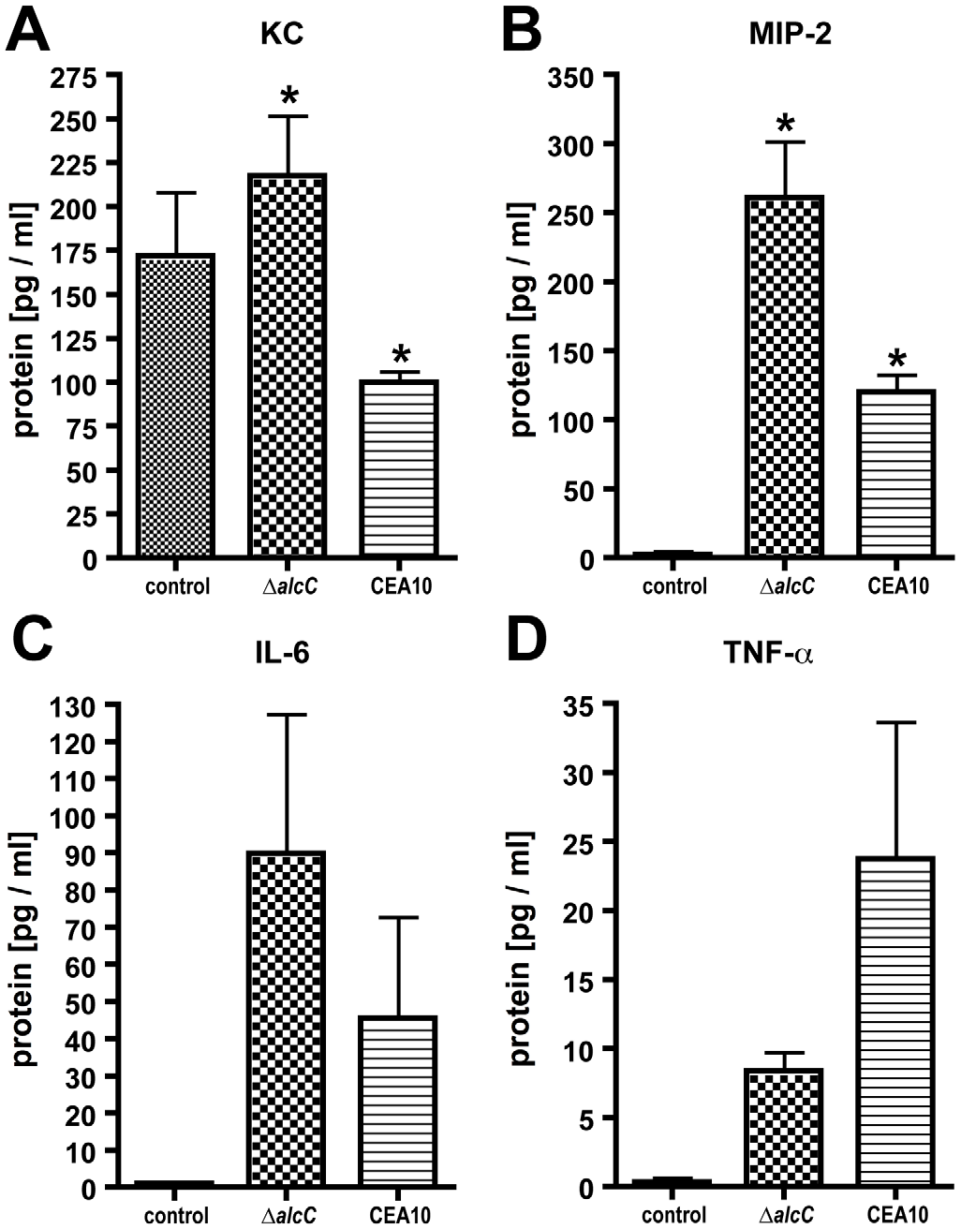
Cytokine production in response to *A. fumigatus* wild type and Δ*alcC* inoculation. (**A**) KC, (**B**) MIP-2, (**C**) IL-6, and (**D**) TNF-α concentrations were determined in BAL fluids on day 3 post inoculation. Significant differences between the two inoculation groups could be observed for KC and MIP-2 protein levels. *p<0.05. Results are the mean and standard error of N = 5 mice.

To further quantify the differences in immune response to Δ*alcC*, we examined the production of cytokines normally associated with neutrophil recruitment in mice (murine IL-8 homologs, KC and MIP2). We observed that protein levels of the two murine neutrophil chemo-attractants KC and MIP-2 were significantly increased in BALs from Δ*alcC* inoculated animals compared with wild type (Figure 10A and B; p<0.05). IL-6 was also slightly elevated while TNF-α protein levels were reduced in comparison to BAL fluids of mice inoculated with the wild type (Figure 10C and D; p>0.05). Altogether, these results indicate that loss of AlcC modulates the immune response of the host to *A. fumigatus* causing increased recruitment of immune effector cells to the site of infection, particularly neutrophils, and associated altered cytokine responses.

### The *A. fumigatus ΔalcC* strain shows reduced *in vivo* fungal growth and causes wild type levels of tissue damage

**Figure 11:**
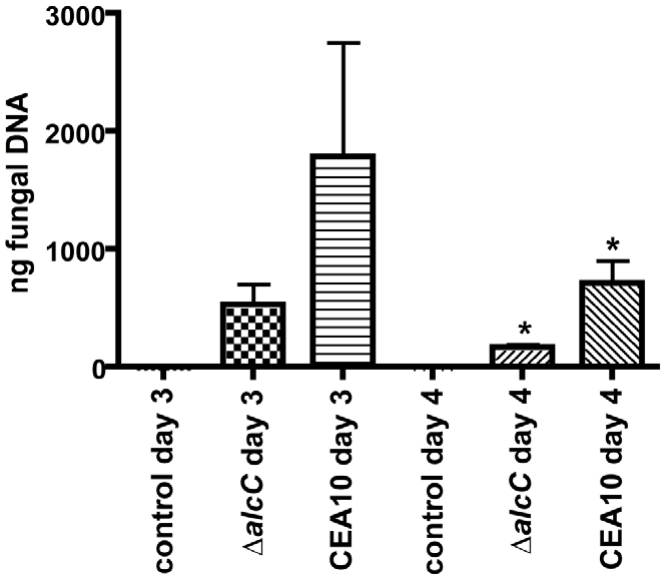
Decreased fungal burden in Δ*alcC* inoculated mice. Outbred CD1 mice were immunosuppressed by subcutaneous injection of triamcinolone (40 mg/kg) 1 day prior to *A. fumigatus* inoculation in a Hinner's inhalation chamber. Fungal burden in the lungs was determined by quantitative real-time PCR based on the 18S rRNA gene of *A. fumigatus*. Data are presented as total fungal genomic DNA normalized to input DNA. The mean and standard error are presented (N = 3 mice for the control group and N = 7 mice for both inoculation groups). * p<0.01.

**Figure 12:**
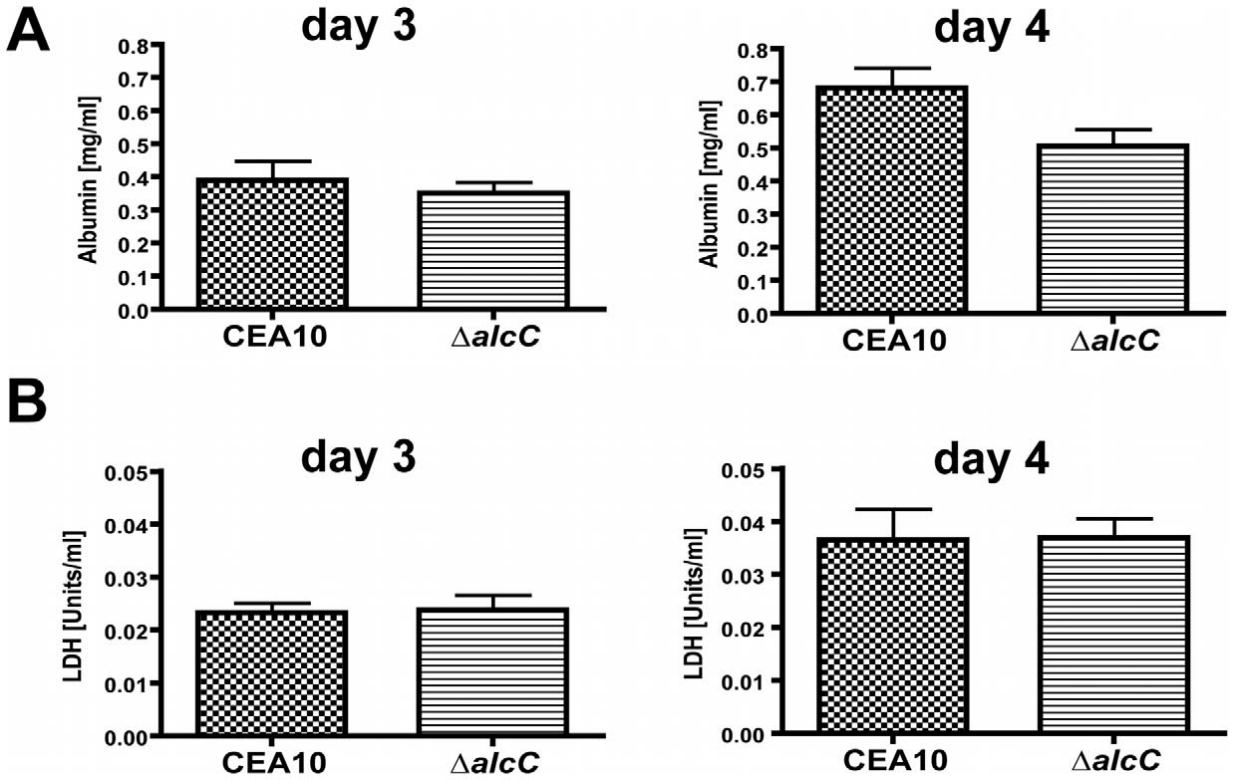
Wild type levels of tissue damage in Δ*alcC* inoculated mice. (**A**) BAL albumin and (**B**) BAL lactate dehydrogenase (LDH) levels in Triamcinolone immunosuppressed CD1 mice (40 mg/kg 1 day prior to infection) were determined on day 3 and 4 p. i. in wild type (CEA10) and Δ*alcC* inoculated mice demonstrating no significant differences between inoculation groups.

The comparison of histopathology between wild type and Δ*alcC* inoculated mice suggested reduced fungal growth by the Δ*alcC* strain in both the chemotherapy and the Triamcinolone murine models (Figure 8). In order to confirm this important observation, we quantified the pulmonary fungal burden on days 3 and 4 p.i. by quantitative RT-PCR. Consistent with the GMS histopathology, qRT-PCR confirmed a reduced pulmonary fungal burden in mice inoculated with Δ*alcC* compared to wild type (Figure 11). In addition, we examined LDH (lactate dehydrogenase) and Albumin release in BAL fluid to determine the degree of tissue damage caused by Δ*alcC* and the wild type strain. Intriguingly, both strains cause the same levels of LDH and Albumin release on day 3 and 4 post inoculation (Figure 12). Collectively, the lower fungal burden, the increased host inflammatory response, and the wild type level of tissue damage in response to Δ*alcC* strongly suggest the *A. fumigatus* alcohol dehydrogenase, AlcC, plays an important role in IPA.

### The increased inflammatory response of the host to the Δ*alcC* strain is not caused by changes in the cell wall

**Figure 13:**
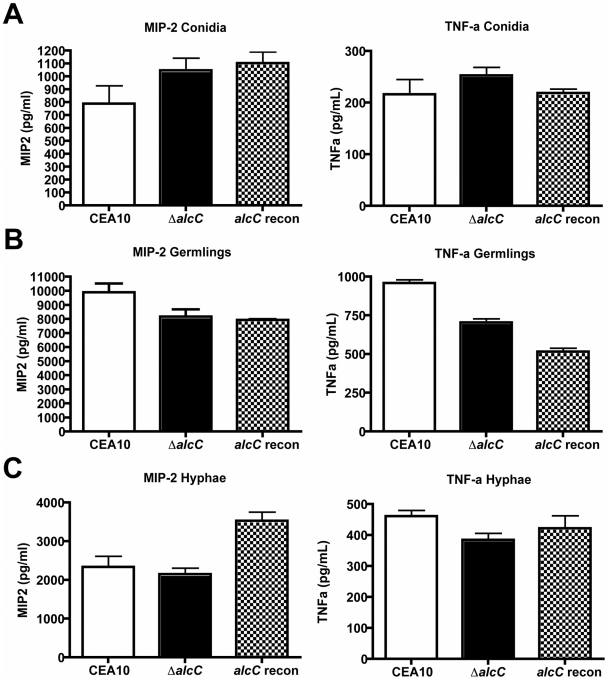
Increased cytokine response is not caused by cell wall changes of the Δ*alcC* strain. Secretion of MIP-2 and TNF-α by bone marrow-derived macrophages (cultured as described in Materials and Methods) incubated for 18 hours with *A. fumigatus* conidia (A), UV irradiated germlings (B), or UV irradiated hyphae (C). Presented are the average concentration and standard deviation in 3 wells per condition of one representative experiment. P>0.05 for all comparisons.

The observed altered host response and reduced fungal burden in animals infected with Δ*alcC* led us to question the mechanism behind these phenotypes. As inflammatory responses to fungal pathogens are often mediated by the fungal cell wall, we tested whether loss of AlcC resulted in unexpected changes to the cell wall of this strain that could account for the *in vivo* phenotypes. Conidia and ultraviolet (UV) irradiated germlings or hyphae from *A. fumigatus* wild type or *ΔalcC* were co-incubated with bone marrow-derived macrophages (BMM∅) and inflammatory cytokine responses were measured (Figure 13). No differences in the secretion of TNF-α or MIP-2 by BMM∅ were observed to any of the tested *A. fumigatus* growth stages with regard to Δ*alcC* or wild type strains. Moreover, no difference in the response to chemical cell wall perturbing agents (caspofungin and congo red) was observed with Δ*alcC* (data not shown). Thus, our data suggest that the increased inflammatory response observed in *ΔalcC* inoculated mice is not caused by changes in the fungal cell wall. Thus, the exact mechanism for the altered pathogenesis in mice inoculated with Δ*alcC* remains to be determined.

## Discussion

Metabolic adaptability and flexibility are important attributes for pathogens to successfully colonize, infect, and cause disease in a wide range of hosts. Importantly, these processes are dynamic, and pathogen and host metabolism are likely to change as the result of the host-pathogen interaction, which alters the localized microenvironment. In this study, we present new insights into the pathogenesis of IPA in commonly used experimental murine models. We present data that confirms previous circumstantial data that suggested that hypoxia may be part of the pathogenesis of IPA [55], [56]. To our knowledge, this is the first confirmation of the occurrence of *in vivo* hypoxic microenvironments in an invasive fungal infection. Our results thereby add a “new” *in vivo* stress to which human fungal pathogens must adapt to cause lethal disease, and it will be intriguing to define the occurrence of hypoxia in other models of human fungal disease.

To determine whether hypoxia actually occurs in the lung during IPA, we used the hypoxia marker, pimonidazole hydrochloride, which is an investigational oncology probe used as a hypoxia-imaging agent in clinical studies to detect reduced oxygen concentrations in animal and human tumors [43], [44], [57], [58], [59]. In our study, we observed that lesions in lungs of mice infected with *A. fumigatus* are hypoxic, as evidenced by the formation of a stable adduct between reduced pimonidazole and host proteins at sites of *A. fumigatus* infection. However, the extent and timing of hypoxia development differed between the immunologically distinct murine models of infection. While hypoxia did occur to some degree in all three models, the chemotherapy model exhibited the least amount of hypoxia in terms of size of the hypoxic area in the lung. This result suggests that the influx and activity of host cells is a strong contributor to the development of hypoxia. However, the persistence of hypoxia in this model, albeit not as extensive as in the other models, also suggests that fungal activities/components contribute to hypoxic lesion. For example, a recent study has observed that *A. fumigatus* can modulate host angiogenesis by secretion of secondary metabolites such as gliotoxin, which may further compromise tissue perfusion and ultimately contribute to coagulative necrosis, and thus limit oxygen delivery to sites of infection [60]. Importantly, though hypoxia was not detected on day +1 or day +2 of infection in any of our models we cannot rule out, and indeed would expect, that conidia and growing hyphal tips experience low oxygen tensions as they are engulfed by various host cells and ultimately penetrate the lung parenchyma and invade into the vasculature. Thus, we conclude that during colonization and subsequent infection, *A. fumigatus* is exposed to a dynamic range of oxygen levels in the lung.

The significance of hypoxia, and the timing and extent to which it occurs during IPA, remain important areas of investigation. One important question that we have started to explore in this study is related to the potential clinical significance of fungal mechanisms of hypoxia adaptation. Previous studies in our and other laboratories with fungal SREBPs have suggested that fungal adaptation to hypoxia is critical for virulence. If true, these mechanisms would become an attractive therapeutic target. However, SREBPs are transcription factors that likely modulate numerous important mechanisms of fungal physiology, and thus it is not currently possibly to attribute the avirulence phenotype of these strains solely to their inability to grow in hypoxia. In general, mechanisms of hypoxia adaptation in human fungal pathogens are unexplored.

Most eukaryotic cells, like *A. fumigatus*, obligatorily use oxygen to carry out many of their biochemical reactions. Oxygen is a key component of energy production where it functions as a terminal electron acceptor in the formation of ATP from glucose during aerobic respiration. When exposed to microenvironments with limited levels of oxygen, many microorganisms utilize fermentation as a potential metabolic mechanism for dealing with the lack of oxygen [34], [35], [36], [37], [38], [39], [40]. Fermentation allows the fungus to replenish sources of NAD^+^ and thus to generate ATP through continued use of glycolysis. Importantly, our interest in hypoxia and fungal pathogenesis began with the discovery of ethanol in BAL samples from *A. fumigatus* infected mice immunosuppressed with our chemotherapeutic model (Figure S1). Thus, in this study, we explored the potential role of ethanol fermentation in *A. fumigatus* hypoxia adaptation and fungal virulence by identifying and characterizing the ethanol fermentation pathway genes in this pathogen.

Our *in vitro* molecular genetic analyses strongly suggest that the main route of ethanol fermentation in *A. fumigatus* is through the pyruvate decarboxylase, PdcA, and subsequent alcohol dehydrogenase, AlcC. Null mutants in both of these genes were unable to produce detectable ethanol *in vitro* under hypoxic conditions. These results are in agreement with observations in *Aspergillus nidulans*, where a *pdcA* deletion strain also fails to produce ethanol [61] and *alcC* activity is induced by hypoxic conditions [62]. However, our results also suggest that ethanol fermentation per se is not required for fungal growth *in vitro* as none of the ethanol fermentation deficient strains displayed any growth differences in *in vitro* normoxic or hypoxic growth conditions. We cannot definitively rule out that a small amount of undetectable ethanol fermentation still occurs in our mutant strains, however, we feel it is more likely that other fermentation pathways exist and/or that sufficient mitochondrial respiration still occurs under the conditions examined to support robust growth.

Despite the persistent growth of the ethanol fermentation deficient stains under hypoxia, the Δ*alcC* displayed a very different phenotype *in vivo* in our murine models of IPA. In our three immunologically distinct models of IPA, no difference in mortality could be observed in mice infected with the wild type and ethanol fermentation mutant strains. However, histopathology of the chemotherapy and Triamcinolone models indicated an increased influx of immune cells and reduced fungal growth in Δ*alcC* inoculated mice. These observations were confirmed by flow cytometry and differential cell counts as well as quantitative fungal burden measurements by qRT-PCR. Although, we observed less fungal burden in Δ*alcC* inoculated mice, the overall lesion size was comparable to wild type caused lesions and both strains caused the same level of tissue damage as measured by LDH and Albumin assays. This result is probably due to the increased influx of neutrophils and macrophages to sites of Δ*alcC* infection. It is tempting to speculate then that Δ*alcC* inoculated mice might succumb to the infection because of the increased host inflammatory response rather than by the invasive growth of the mold. The decrease in fungal burden in mice infected with the Δ*alcC* strain might suggest that this response is partially effective at limiting fungal growth, but with collateral damage to the host that results in mortality. Of note, Mehrad *et al.* recently observed that an overproduction of KC results in a lower fungal burden and higher levels of neutrophil recruitment in a murine model of IPA, which leads to more resistance to *A. fumigatus* infections [63]. As we observed increases in KC and MIP2 levels in response to Δ*alcC* with a concomitant decrease in fungal burden, it may be possible that the increased inflammatory response to Δ*alcC* is at least partially antifungal.

The *in vivo* phenotype of Δ*alcC* raises some intriguing questions about the mechanism behind the observed increase in host inflammatory response and subsequent reduction in fungal burden. Previous observations have indicated that ethanol is a potent immunosuppressive agent, and thus it seems reasonable to hypothesize that loss of ethanol production at the site of infection at least partially explains the observations with Δ*alcC* [48], [49], [52], [64], [65], [66], [67], [68]. Acute and chronic ethanol exposures have been shown to alter the immune response to both bacterial and viral pathogens [69], [70], [71]. Ethanol decreases clearance of pneumococci and *Klebsiella* species from the lungs of ethanol-fed mice, which is mainly due to an impaired response of the phagocytic cells [72]. With regard to fungal pathogens, Zuiable *et al.* observed that human blood monocytes incubated with ethanol have impaired killing of *Candida albicans* [51]. Along these lines of thinking it may be possible that *A. fumigatus* is able to partially suppress localized host immune responses by utilizing ethanol fermentation in response to hypoxic microenvironments during IPA. However, to confirm this hypothesis, more sensitive detection methods for the localized and low levels of ethanol produced at the site of *A. fumigatus* infection are needed.

Currently, ethanol detection in our murine models is inconsistent as exhibited by our initial experiment with BALs and ^1^H-NMR. BALs only sample the airway and do not sample localized infection sites located in the lung parenchyma so it is potentially not surprising that this method may not consistently detect a small molecule in the lung such as ethanol. To overcome this potential limitation, we attempted to utilize whole lung homogenates and two different ethanol detection methods including an enzymatic based approach and a GC-MS approach. Unfortunately, either the complexity of the samples, the chemical nature of ethanol itself, or the metabolism at the site of infection prevented reproducible detection of ethanol. Thus, currently, we cannot directly attribute the increased inflammatory response observed with Δ*alcC* to loss of ethanol production. However, development of more sensitive detection methods is underway in our laboratory.

It is intriguing to note that the possibility of fermentation being important for hypoxic growth during fungal infections is further supported by the finding of ethanol in cerebral tissue of rats infected with *C. neoformans* [73]. Moreover, in support of the hypothesis that it is at least a secreted factor that is affecting the host response to Δ*alcC*, UV killed wild type and Δ*alcC* strains at 3 distinct growth phases do not exhibit a difference in pro-inflammatory responses *ex vivo* with bone marrow derived macrophages (Figure 13). Thus, the most likely culprit for an altered inflammatory response, the fungal cell wall, appears to not be altered in Δ*alcC*. Future studies will continue to probe the mechanism behind the reduced fungal growth and increased inflammatory response of Δ*alcC.*

Altogether, in this study we present the first *in vivo* observations of hypoxic microenvironments during an invasive pulmonary fungal infection and shed light on how the mold *A. fumigatus* adapts to low oxygen environments to cause disease. These results, along with other published data from our laboratory, continue to support the hypothesis that hypoxia adaptation and growth is an important component of the pathogenesis of IPA [29], [31], [74]. Our results further emphasize the dynamic and complex interactions that occur between fungi and their hosts during an invasive pulmonary fungal infection. Future studies will continue to explore the effects of infection localized microenvironment stresses on invasive pulmonary aspergillosis pathogenesis. It will be intriguing to learn if other human fungal pathosystems also involve significant levels of hypoxia at sites of infection and whether ethanol fermentation pathway mutants also alter the host response.

## Materials and Methods

### Strains and media

*A. fumigatus* strain CEA17 (gift from Dr. J.P. Latgé, Institut Pasteur, Paris, France) was used to generate the *pdcA* (AFUB_038070; *ΔpdcA*::*A. parasiticus pyrG pyrG1*), *pdcB* (AFUB_096720; *ΔpdcB*::*A. parasiticus pyrG pyrG1*), *pdcC* (AFUB_062480; *ΔpdcC*::*A. parasiticus pyrG pyrG1*), and *alcC* (AFUB_053780; *ΔalcC*::*A. parasiticus pyrG pyrG1*) null mutant strains. *A. fumigatus* strain CEA17 is a uracil-auxotrophic (*pyrG1*) mutant of *A. fumigatus* strain CEA10 [75], [76]. In this study, we used CEA10 (CBS144.89) (gift from Dr. J.P. Latgé, Institut Pasteur, Paris, France) as the wild type strain in all experiments except the ^1^H-NMR metabolite profiling experiment which utilized strain AF293, *ΔpdcA*, *ΔpdcB*, *ΔpdcC*, *ΔalcC*, and the ectopic complemented control strains, *pdcA* recon (*ΔpdcA*::*A. parasiticus pyrG* + *pdcA*) and *alcC* recon (*ΔalcC*::*A. parasiticus pyrG* + *alcC*). All strains were routinely grown in glucose minimal medium (GMM) with appropriate supplements as previously described [77] at 37°C. To prepare solid media 1.5% agar was added before autoclaving.

### Strain generation

Generation of the *pdc* null mutants and the *alcC* null mutant in *A. fumigatus* strain CEA17 were accomplished by replacing the ORF of the target genes with *A. parasiticus pyrG*. The replacement construct was generated by cloning a sequence homologous to the gene locus into plasmid pJW24 (donated by Dr. Nancy Keller, University of Wisconsin – Madison). Homologous sequences, each ∼1 kb in length and 5′ and 3′ of the gene coding sequence, were cloned to flank *A. parasiticus pyrG* in pJW24. The resulting plasmids, pPDCAKO, pPDCBKO, pPDCCKO, and pALCCKO, were used as templates to amplify a disruption construct (3.6–4.7 kb) for use in fungal transformation.

To complement the *ΔpdcA* and *ΔalcC* strains the genes were amplified using genomic DNA of CEA10 as template and primers ∼1 kb 5′ and ∼500 bp 3′ of the gene coding sequence. The PCR products were cloned in front of the hygromycin B resistance gene into plasmid pBC-hygro (Silar 1995, obtained from the Fungal Genetics Stock Center, Dr. Kevin McCluskey) using SpeI and NotI restriction sites [78], [79]. The resulting plasmids, pBC-hyrgo-PDCA and pBC-hygro-ALCC, were used as template to amplify complementation constructs (∼7.4 kb), which were used in a fungal transformation and selection was for colonies able to grow on media containing 150 μg/ml hygromycin B. The primers utilized in vector construction are presented in Table S1.

Standard fungal protoplast transformation was used to generate mutant and reconstituted strains as previously described [31]. Transformants were initially screened by PCR to identify potential homologous recombination events at the gene locus using primers designed to amplify only the mutated gene locus (Table S1). Single conidia of each transformant were prepared and screened by PCR to eliminate the chance of heterokaryons. Homologous recombination was confirmed by Southern analysis with the digoxigenin labeling system (Roche Molecular Biochemicals, Mannheim, Germany) as previously described [80].

### Hypoxic cultivation

Strains were grown on or in GMM at 37°C. Normoxic conditions were considered general atmospheric levels within the lab (∼21%). For hypoxic conditions two different devices were used, a Biospherix C-Chamber with O_2_ levels controlled by a PRO-Ox controller and CO_2_ levels controlled with PRO-CO_2_ controller (Biospherix, Lacona, NY, USA) and an INVIVO_2_ 400 Hypoxia Workstation (Ruskinn Technology Limited, Bridgend, UK). For these experiments, the O_2_ set point was 1% and the CO_2_ set point was 5%. Oxygen levels were maintained with 94% N_2_ and a gas regulator. Colony growth was quantified as previously described [31].

### Pyruvate decarboxylase activity assay

For normoxic samples, strains were grown in GMM with 1x10^6^ conidia/ml, 300 rpm, at 37°C for 16 hrs. 25 ml of the normoxic culture were mixed with 15 ml of fresh GMM and incubated for an additional 24 hrs under hypoxic conditions (130 rpm, 37°C). Mycelia of normoxic and hypoxic cultures were harvested, rinsed twice with distilled water, transferred to 2 ml screw cap tubes with 0.1 mm glass beats, immediately frozen in liquid nitrogen and weighed. After adding 1 ml of extraction buffer (100 mM KH_2_PO_4_, 2 mM MgCl_2_, and 1 mM DTT), the samples were twice placed in a mini beadbeater (Biospec products, Bartlesville, OK, USA) for 30 sec with 5 min on ice in between. After centrifugation (13,000 rpm, 20 min, 4°C) the cell free extracts were transferred to new, cold reaction tubes and kept on ice until use. The protein concentration of the cell free extracts was defined by using the Coomassie Plus – The Better Bradford Assay Kit (Pierce, Rockford, IL, USA) following the method recommended by supplier.

Enzyme activity was determined using a method adapted from Lockington *et al.* 1997 [61]. The assay volume was adjusted to 200 µl for use in 96-well plates. 25 µl cell free extract (sample and control) or 25 µl extraction buffer (blank) were added to wells in duplicates and then 175 µl of the sample mix (50 mM histidine-HCl, 0.35 mM MgCl_2_, 0.35 mM TPP, 67 mM pyruvate, 6 U yeast ADH, 0.5 mM fresh NADH, added up with water to 175 µl) were added to the samples and the blank, and 175 µl of the control mix (sample mix without pyruvate) were added to the cell free extract as controls. The rate of decrease in absorbance at 340 nm was followed in a Spectramax Plus (Molecular Devices, Sunnyvale, CA, USA), measuring every 10 sec, at 37°C over 5 min after mixing for 1 sec.

The pyruvate decarboxylase activity was calculated as described by http://cmbe.engr.uga.edu/assays/pyruvatedecarboxylase.pdf. The calculation had to be adjusted to the reduced length of the light path in the 96-well plate by multiplying the molar extinction coefficient for NADH (6.22 L/mmol for a path length of 1 cm) with 0.788. Experiments were done in three separate biological replicates and the mean and standard error calculated with Prism software version 5.0b (GraphPad Software Inc.).

### Ethanol detection

To detect ethanol in the culture supernatant of *in vitro* fungal cultures, high performance liquid chromatography (HPLC) was performed using a Shimadzu system (Kyoto, Japan), consisting of a solvent delivery module, a low pressure gradient pump unit, a degasser, an autoinjector, a column oven and a refractive index. The column used for the analytical separation was the Aminex Fermentation Monitoring column (150 mm×7.8 mm, BioRad, Hercules, CA). The mobile phase consisted of 0.001 M H_2_SO_4_, the flow rate was 0.8 ml/min, the column temperature was 60°C, and the sample injection volume was 25 µl. As external standard ethanol solutions with known concentration (2, 1, 0.5, 0.1, 0.05, 0.01 (v/v) %) were used in every experiment and a standard curve was generated and used to determine concentration. Data was normalized to mycelial dry weight.

In addition, a Shimadzu (Kyoto, Japan) QP2010 GC/MS with an electron ionization (EI) source was used for metabolite separation and identification. A 30 m 0.25 mm id 0.25 um film thickness, RTX-5MS (5% Diphenyl - 95% dimethyl polysiloxane) fused silica capillary column from Restek (Bellefonte, PA) was used for all separations. The GC column was temperature programmed as follows: 5 min isothermal at 100°C, then raised at 20°C/minute to 120°C, and held for 30 seconds. Helium gas served as the carrier gas at a flow rate of 0.73 ml/min. Split injections were performed at a 1 to 20 ratio. The injection port was held constant at 200°C. The interface temperature was set at 200°C and the ion source at 200°C. EI fragmentation was performed scanning from 40 to 400 at 0.2 seconds/scan. The instrument was calibrated with Perfluorotributylamine (PFTBA) prior to analysis. Standards of ethanol were analyzed for rention time and a response curve using a 3 point serial dilution. These response curves were used to calculate detected compounds in each sample. One micro-liter of each sample and standard was used for analysis. Identification was matched to NIST 21 and NIST 107 libraries commercially purchased as well as secondary confirmation with standards, previously mention, purchased from Sigma (Saint Louis, MO).

For the culture samples, strains were grown as described above in the pyruvate decarboxylase assay description. After 24/48/72/96 hrs 2 ml of the culture supernatant were transferred into sterile reaction tubes on ice, and filtered through a Millipore membrane filter (0.45 µm, Millipore, Yonezawa, Japan) into HPLC vials (Sun Sri, Rockwood, TN). Experiments were done in three biological replications.

### Isolation of total RNA and transcriptional profiling

Conidia from freshly harvested GMM plates were inoculated in 5 ml GMM in a 6-well plate to a concentration of 1×10^7^ conidia/ml. Cultures were grown aerobically for 24 h. For normoxic growth, cultures were maintained in atmospheric conditions. For hypoxic growth, cultures were placed in the hypoxic chamber for 24 h. Fungal mats were flash frozen in liquid nitrogen and lyophilized prior to disruption using a bead beater. To assess fungal gene expression *in vivo*, the triamcinolone immunosuppression model was utilized as described below. Mice were sacrificed on day 3 and 4 post inoculation, and lungs were harvested and immediately frozen in liquid nitrogen. Samples were freeze-dried and homogenized with glass beads on a Mini-Beadbeater (BioSpec Products, Inc., Bartlesville, OK, USA). Total RNA was extracted using TRIsure Reagent (Bioline, Tauton, MA, USA) according to the manufacturer's instructions. After treatment with DNase I (Ambion, Austin, TX, USA), 500 ng of total RNA were used to generate first-strand cDNA with the reverse transcriptase kit (Qiagen, Hilden, Germany). Real-time PCR assays were performed with 20 µl reaction volumes that contained 1x iQ SYBR green master mix (Biorad, Hercules, CA, USA), 0.2 µM of each primer (Table S1), and 2 µl of a 1∶5 dilution of the cDNA using a Bio-Rad MyiQ single Color real-time PCR detection System with iCycler. No-RT controls for each primer set were also assayed to confirm that no DNA contamination was present, respectively. Real-time PCRs were performed in triplicates, and the expression levels of all genes of interest were normalized to ß–tubulin levels or *tefA* (translation elongation factor alpha subunit) levels for *in vivo* experiments. The thermal cycling parameters consisted of a 3-min Taq polymerase hot start at 95°C, followed by template amplification of 40 cycles of 95°C for 10 sec, 58°C for 30 sec. Fluorescence was measured during the annealing/extension step (58°C) and a disassociation analysis (melting curve) was performed to confirm that a single amplified product was present. Following amplification, data was analyzed with the Bio-Rad iQ5 2.0 Standard Edition Optical System Software. The ΔΔCt method of analysis was used to determine fold changes of gene expression in the mutants relative to the wild type CEA10 strain.

### Murine models of invasive pulmonary aspergillosis

The virulence of the *A. fumigatus* strains was tested in three immunologically distinct murine models of invasive pulmonary aspergillosis. All animals were housed five per cage in an environment with HEPA filtered air, autoclaved food at libitum, and prophylactic treatment with antibiotic water containing clindamycin (150 mg/ml), vancomycin (1 mg/L) and gentamicin (100 mg/ml). CD1 male and female mice, 6–8 weeks old were used in all experiments for the triamcinolone and chemotherapeuatc murine models. Mice were obtained from Charles River Laboratories (Raleigh, NC) or from a breeding colony located in the Animal Resources Center (ARC) at Montana State University. For the Chronic Granulomatous Disease murine model, 6–8 week old mice with a null allele corresponding to the X-linked gp91^phox^ component of NADPH oxidase (B6.129S6-Cyb^btm1Din^) were bred in the ARC at Montana State University [45].

For the triamcinolone (corticosteroid) model mice were immunosuppressed with a single dose of Kenalog (Bristol-Myers Squibb Company, Princeton, NJ, USA) injected subcutaneously (s.c.) at 40 mg/kg 1 day prior to inoculation. For the chemotherapy model mice were immunosuppressed with intraperitoneal (i.p.) injections of cyclophosphamide (Baxter Healthcare Corporation, Deerfield, IL, USA) at 175 mg/kg 2 days prior to inoculation and with Kenalog injected subcutaneously (s.c.) at 40 mg/kg 1 day prior to inoculation. On day 3 post-inoculation (p.i.), repeat injections were given with cyclophosphamide (150 mg/kg i.p.) and on day 6 p.i. with Kenalog (40 mg/kg s.c.).

For the detection of hypoxia *in vivo*, 15 unanesthetized mice inhaled 40 ml of an aerosolized suspension of 1×10^9^ conidia/ml of *A. fumigatus* strain CEA10. 6 uninfected control mice inhaled 40 ml of aerosolized 0.01% Tween 80 in a Hinners inhalational chamber for 45 min as previously described [81]. One *A. fumigatus* infected mouse was sacrificed immediately after infection, lungs were removed, homogenized and the number of CFU was determined (∼1×10^4^ conidia per mouse). After hypoxyprobe injection (see below), mice were sacrificed at set time points after *A. fumigatus* challenge and lungs were processed for hypoxyprobe immunohistochemistry.

For survival studies and histopathology, 10 mice per *A. fumigatus* strain (CEA10, *ΔpdcA*, *pdcA* recon., *ΔalcC*) either inhaled 40 ml of an aerosolized suspensions of 1x10^9^ conidia/ml (control mice inhaled 40 ml of aerosolized 0.01% Tween 80) or the animals were inoculated intranasally with 1×10^6^ conidia in 25 µl and monitored twice a day. Infection inoculum was prepared by growing the *A. fumigatus* isolates on GMM agar plates at 37°C for 3 days. Conidia were harvested by washing the plate surface with sterile phosphate-buffered saline 0.01% Tween 80. The resultant conidial suspension was adjusted to the desired concentration by hemacytometer count.

Mice were observed for 14 days after *A. fumigatus* challenge. Any animals showing distress were immediately sacrificed and recorded as deaths within 24 hrs. No mock infected animals perished in either murine model in all experiments. Lungs from all mice sacrificed on different time points during the experiment were removed for fungal burden assessment, infiltrate and cytokine analysis as well as histopathology. Animal experiments were all repeated in duplicate.

### Ethics statement

This study was carried out in strict accordance with the recommendations in the Guide for the Care and Use of Laboratory Animals of the National Institutes of Health. The animal experimental protocol was approved by the Institutional Animal Care and Use Committee (IACUC) at Montana State University (Federal-Wide Assurance Number: A3637-01).

### Histopathology

For histopathology, CD1 mice were inoculated as described above, and sacrificed at set time points after *A. fumigatus* challenge. When mice were sacrificed, lungs were removed on that day. Lung tissue was fixed in 10% phosphate-buffered formalin, embedded in paraffin, sectioned at 5 µm, and stained with hematoxylin and eosin (H&E) or Gomori methenamine silver (GMS) by using standard histological techniques. Microscopic examinations were performed on a Nikon Eclipse 80i microscope and imaging system (Nikon Instruments Inc., Melville, NY, USA). A total of 3 mice were examined at each respective time point.

### ^1^H-NMR metabolite profiling of broncheoalveolar lavage fluid

The chemotherapeutic murine model of IPA was utilized in these experiments. Mice were inoculated in the Hinner's chamber with either 0.01% Tween 80 or *A. fumigatus* wild type strain AF293. A total of 10 mice were used in each treatment group. On day +3 post infection, BALs were performed with each BAL a final total volume of ∼1.5 ml. Deuterated water was added to 600 μL of each BAL to provide a field frequency lock and an internal standard of 0.03% 3-(Trimethylsilyl)-Propionic acid-D_4_ sodium salt (TSP) was added to each sample to provide a chemical shift reference at 0 ppm. For ^1^H-NMR, a one-pulse sequence was used with a 2-second pre-saturation pulse and 7-second repetition time. The resulting one-dimensional spectra were compared using MestReC NMR analysis software (Mestrelab Research) to monitor the presence and absence of identifiable metabolites.

### Hypoxyprobe – treatment, staining and immunohistological identification

Mice were intravenously injected with hypoxyprobe at a dose of 60 mg/kg weight of the mouse (Hypoxyprobe Inc., Burlington, MA, USA). After 60 to 90 min, mice were sacrificed by pentobarbital anesthesia (100 µg/g body weight) followed by exsanguination. The left lung of each mouse was filled with OCT (frozen tissue matrix) and after embedding in OCT immediately frozen in liquid nitrogen. The lungs were cryosectioned into 5 µm sections and stored at −80°C until stained. After thawing, the sections are fixed in cold acetone (4°C) for 15 min, followed by washing the sections (PBS, 2×5 min) and blocking with normal serum block (NSB: PBS +10% goat serum +1.25% mouse serum) at RT. After 30 min, sections were washed and incubated overnight at 4°C with the mouse monoclonal antibody FITC-Mab1 (Hypoxyprobe-1 Plus Kit, Hypoxyprobe Inc., Burlington, MA, USA) diluted 1∶400 in NSB and with a rabbit polyclonal antibody to *Aspergillus* (Abcam Inc., Cambridge, MA, USA). *Aspergillus* isotype control slides were incubated only with FITC-Mab1 and hypoxyprobe isotype control slides only with the *Aspergillus* antibody. Isotype controls are a measure of unspecific staining of the secondary antibody. After another wash, sections were incubated for 60 min at room temperature with DyLight 594-conjugated mouse Anti-FITC (Jackson ImmunoResearch Laboratories, West Grove, PA) and AlexaFluor488-conjugated goat Anti-rabbit (Invitrogen, Carlsbad, CA, USA) diluted 1∶400. After another washing step, prolong Gold antifade reagent with DAPI (Invitrogen, Carlsbad, CA, USA) was added to each section. Microscopic examinations were performed on a Zeiss Axioscope 2-plus microscope and imaging system (Zeiss, Jena, Germany). For each time point, a total of 2 to 4 mice were examined and experiments were repeated in triplicate.

### Evaluation of pulmonary infiltrate by flow cytometry and cell differentiation

Broncheoalveolar lavages (BALs) were performed by intratracheal instillation and extraction of 3 ml 1x PBS. Total lung lavage cell numbers were determined by hemacytometer count, spun onto glass slides, and stained with Diff-Quick (Fisher Scientific, Pittsburgh, PA, USA) for differential counting. For flow cytometry, BAL cells were centrifuged and resuspended in phosphate-buffered saline with 2% calf serum and an anti-mouse Fc receptor antibody (Trudeau Institute, Saranac Lake, NY, USA) to a concentration of approximately 10^7^ cells/ml. The cells were then stained with a mixture of fluorophore-conjugated antibodies against mouse GR-1 (APC-Cy7) (BD Pharmingen, San Diego, CA, USA), F4/80 (PE-Cy7) (eBioscience, San Diego, CA, USA), CD11b (AlexaFluor700) (BioLegend, San Diego, CA, USA), and CD11c (APC) (purified from the hamster cell line N418 (ATCC, Manassas, VA, USA) and fluorophore-conjugated using the AlexaFluor633 protein labeling kit (Invitrogen, Carlsbad, CA, USA)) and then examined on a BD LSR flow cytometer (BD Biosciences, San Jose, CA, USA). Analysis of cytometry data was performed with FlowJo software Version 8.8.7 DMG and numbers of relevant cell types were determined by combining flow cytometry data (percentage of a given cell type) with BAL cell counts. Data presented are the mean and standard error of N = 5 mice at each time point.

### Detection of cytokines, chemokines and other signal proteins in BAL fluids

The BD Cytometric Bead Array Mouse Inflammation Kit (BD Biosciences, San Jose, CA, USA) was used according to the manufacturers instructions to quantitatively measure IL-6, IL-10, MCP-1, IFN-γ, TNF-α, and IL-12p70 protein levels in mouse BAL fluids utilizing a FACSCalibur flow cytometer (Becton Dickinson, Mountain View, CA, USA). IL-17, MIP-2, KC, and VEGF levels in mouse BAL samples were determined using the mouse cytokine/chemokine Milliplex Map Kit (Millipore Corporation, Billerica, MA, USA) according to the manufacturers instructions and then examined and analyzed on the BioPlex 200 System (Bio-Rad, Hercules, CA, USA). Data presented are the mean and standard error of N = 5 mice at each time point.

### Determination of LDH and Albumin levels in BAL fluids

*In vivo* lung tissue damage was determined by measurement of LDH and Albumin levels in mouse BAL samples by using a LDH assay (CytoTox 96 Non-Radioactive Cytotoxicity Assay, Promega, Madison, WI, USA) and an albumin assay (Albumin (BCG) Reagent Set, Eagle Diagnostics, Cedar Hill, TX, USA) according to the manufacturers' instructions.

### Determination of *in vivo* fungal burden

To assess fungal burden in lungs, the triamcinolone immunosuppression model was utilized as described above. Mice were sacrificed on day 3 and 4 post inoculation, and lungs were harvested and immediately frozen in liquid nitrogen. Samples were freeze-dried, homogenized with glass beads on a Mini-Beadbeater (BioSpec Products, Inc., Bartlesville, OK, USA), and DNA extracted with the E.N.Z.A. fungal DNA kit (Omega Bio-Tek, Norcross, GA, USA). Quantitative PCR was performed as described previously [82].

### *In vitro* cytokine response

Bone marrow cells were eluted from the tibias and femurs of 8–12 week old C57BL/6 mice, lysed of red blood cells, and cultured in RP20 (RPMI, 20% FCS, 5 mM HEPES buffer, 1.1 mM L-glutamine, 0.5 U/ml penicillin, and 50 mg/ml streptomycin) supplemented with 30% (v/v) L929 cell supernatant (source of M-CSF). Bone marrow cells were plated in a volume of 20 ml at a density of 2.5×10^6^ cells/ml in 10 ml Petri dishes. The medium was exchanged on day 3. Adherent bone marrow-derived macrophages (BMMØs) were harvested on day 6. Cells were washed and plated in 0.2 ml RP10 at a density of 5×10^5^ cells/ml in 96 well plates and stimulated for 18 hours with conidia (5×10^5^ per well), UV irradiated germlings (10^5^/well), or UV irradiated hyphae (2×10^4^/well) prepared as described before [83]. After 18 hours co-culture supernatants were collected for ELISA. Commercially available ELISA kits for TNF (BD Biosciences, San Jose, CA, USA) and MIP-2 (R&D Systems, Minneapolis, MN, USA) were used according to the manufacturers' instructions.

## Supporting Information

Figure S1 Representative 400 MHz ^1^H-NMR spectra of broncheoalveolar lavage (BAL) fluid from a mouse with a day +3 *A. fumigatus* pulmonary infection (top spectra) or an uninfected mouse (bottom spectra). Known metabolites are labeled on the infected mouse spectra. Substantial amounts of ethanol are seen in the infected mouse samples.
(TIF)

Figure S2 Hypoxic microenvironments at the site of *A. fumigatus* infection. Single color channel pictures were merged to show overlapping localization of fungal tissue (green), hypoxia (red), and inflammation (DAPI stained nuclei).
(TIF)

Figure S3 Representative histopathology of X-CGD mice inoculated with wild type (CEA10) or Δ*alcC* conidia using a Hinner's inhalational chamber. Mice were euthanized on indicated days p.i., lungs removed, fixed, and stained with hematoxylin and eosin (H&E) or Gommori's methenamine silver (GMS) stain. Lung histopathology showed strongly reduced fungal growth of both strains with simultaneous massive inflammation. No difference could be observed between infection groups. Bar = 100 µm.
(TIF)

Figure S4 Flow cytometry Dot plot for F4/80 and CD11c.
(TIF)

Figure S5 Flow cytometry Dot plot for GR-1 and CD11b.
(TIF)

Table S1 Nucleotide sequences of primers used for deletion and complementation strain constructions as well as real-time PCR.
(XLS)
