# Supplementary figures and images for: In vivo Hypoxia and a Fungal Alcohol Dehydrogenase Influence the Pathogenesis of Invasive Pulmonary Aspergillosis
Source: PLoS Pathog. 2011 Jul 21;7(7):e1002145. doi: 10.1371/journal.ppat.1002145 (PMC3141044; doi:10.1371/journal.ppat.1002145)

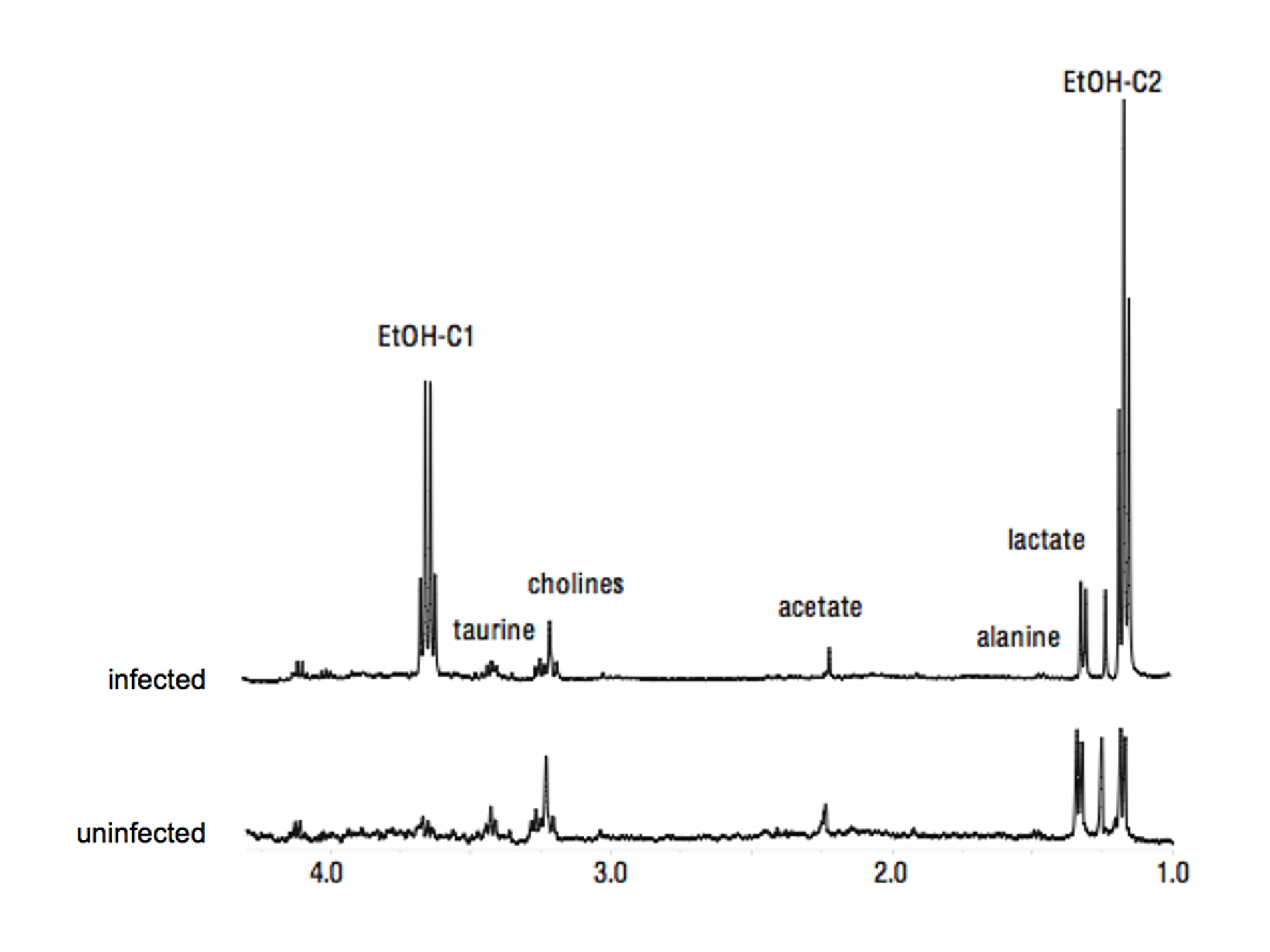

Supplement: Figure S1 — Representative 400 MHz 1H-NMR spectra of broncheoalveolar lavage (BAL) fluid from a mouse with a day +3 A. fumigatus pulmonary infection (top spectra) or an uninfected mouse (bottom spectra). Known metabolites are labeled on the infected mouse spectra. Substantial amounts of ethanol are seen in the infected mouse samples. (TIF) [file ppat.1002145.s001.tif]

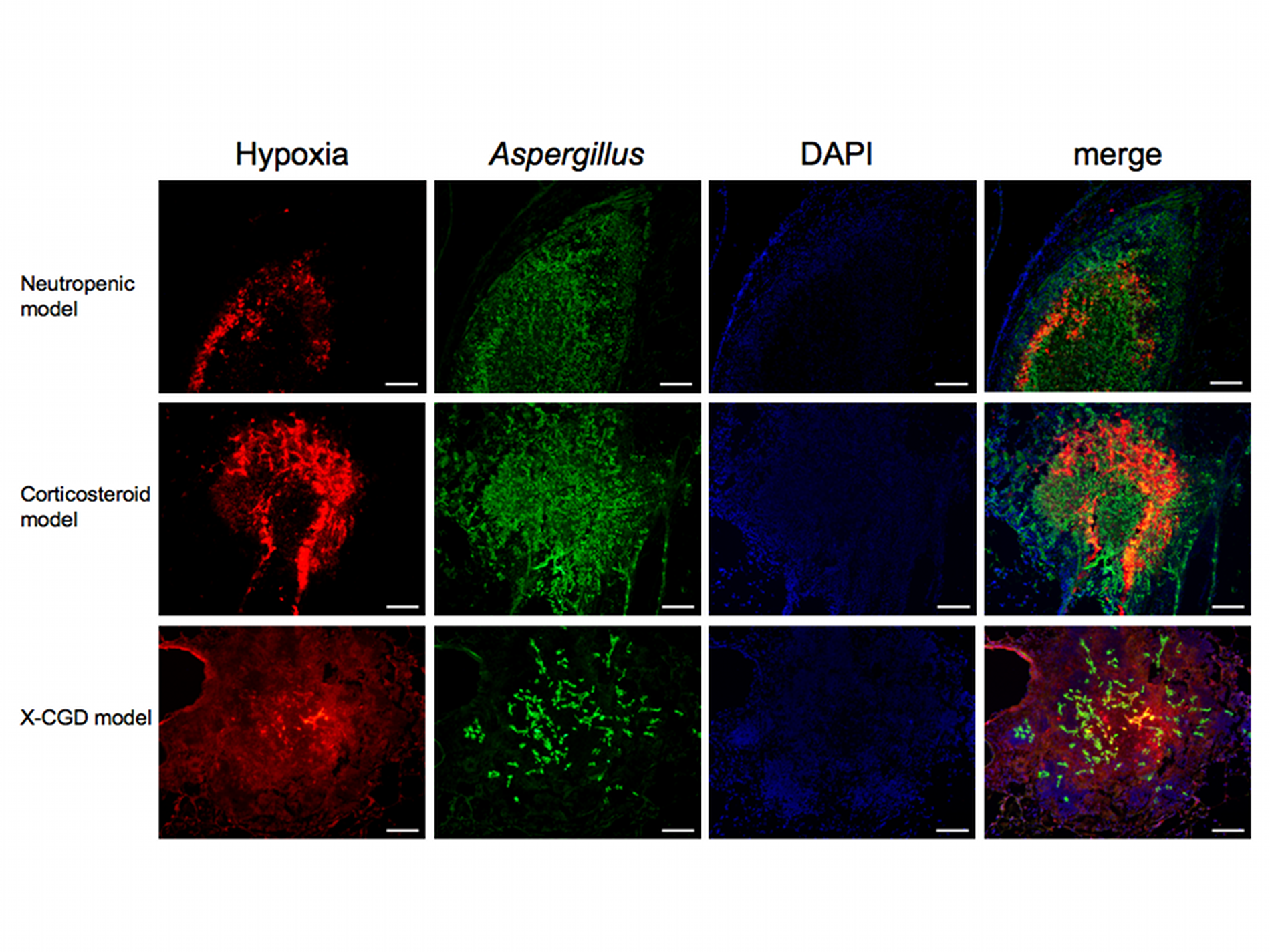

Supplement: Figure S2 — Hypoxic microenvironments at the site of A. fumigatus infection. Single color channel pictures were merged to show overlapping localization of fungal tissue (green), hypoxia (red), and inflammation (DAPI stained nuclei). (TIF) [file ppat.1002145.s002.tif]

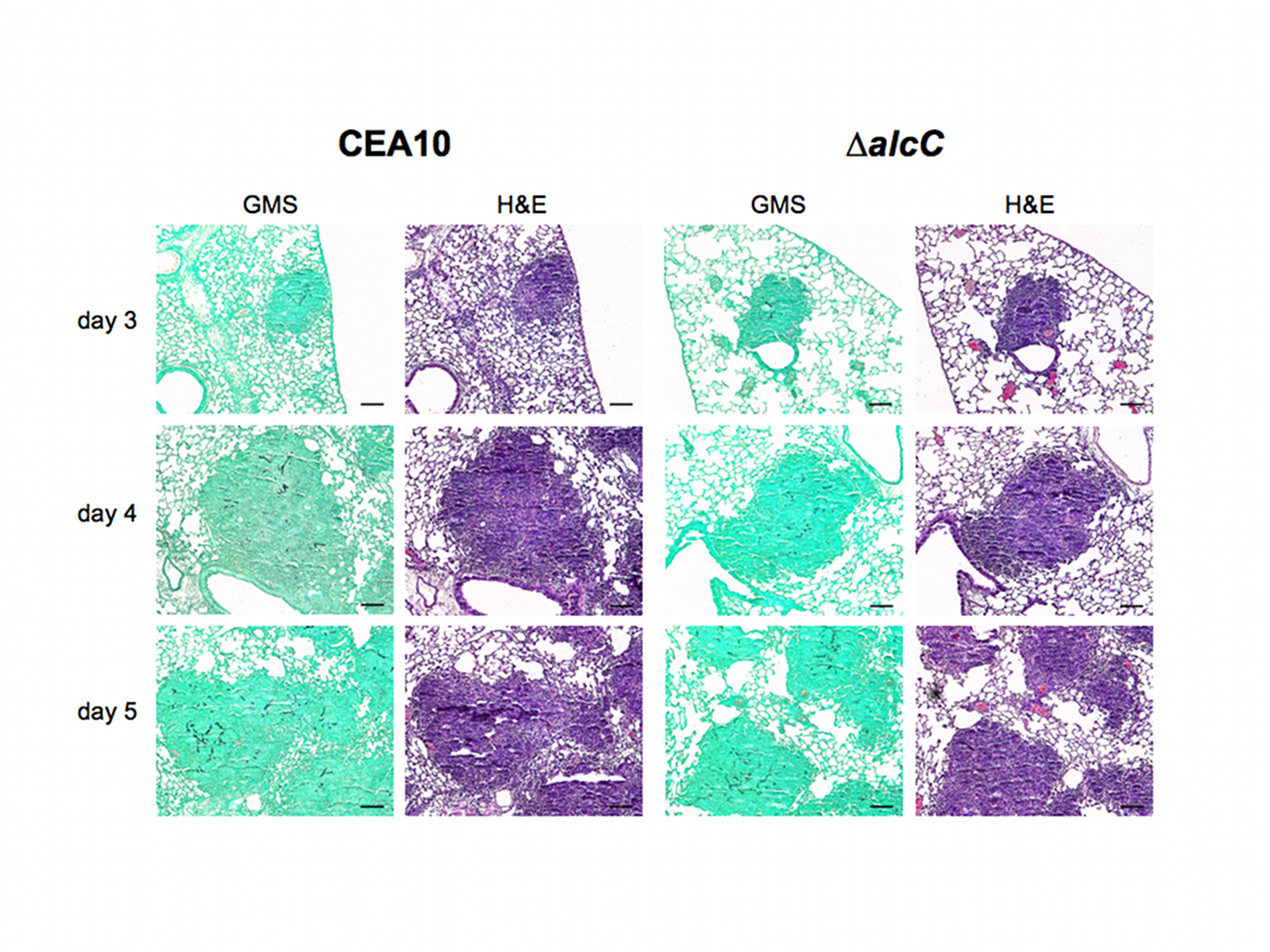

Supplement: Figure S3 — Representative histopathology of X-CGD mice inoculated with wild type (CEA10) or ΔalcC conidia using a Hinner's inhalational chamber. Mice were euthanized on indicated days p.i., lungs removed, fixed, and stained with hematoxylin and eosin (H&E) or Gommori's methenamine silver (GMS) stain. Lung histopathology showed strongly reduced fungal growth of both strains with simultaneous massive inflammation. No difference could be observed between infection groups. Bar = 100 µm. (TIF) [file ppat.1002145.s003.tif]

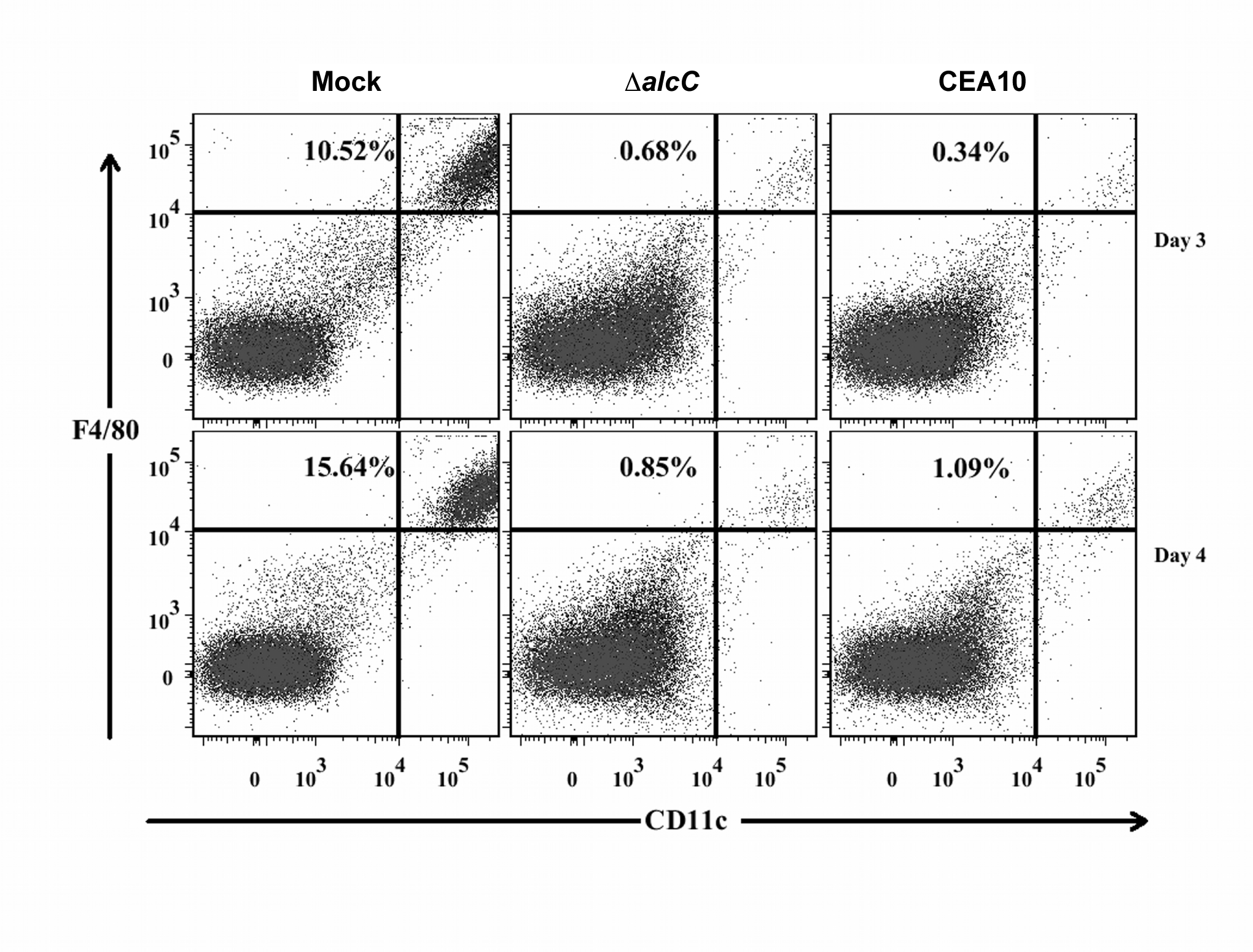

Supplement: Figure S4 — Flow cytometry Dot plot for F4/80 and CD11c. (TIF) [file ppat.1002145.s004.tif]

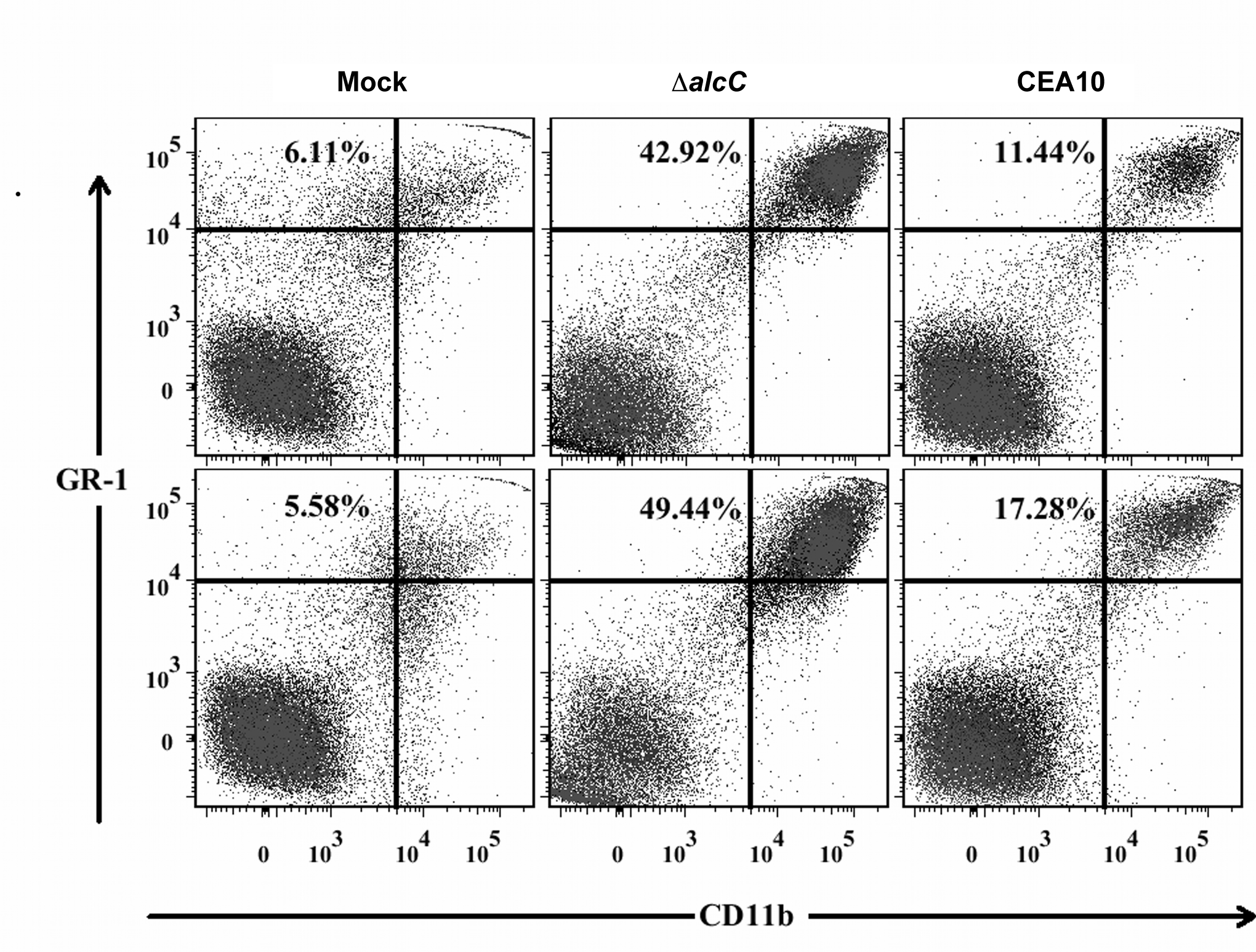

Supplement: Figure S5 — Flow cytometry Dot plot for GR-1 and CD11b. (TIF) [file ppat.1002145.s005.tif]
